# Slow but Steady: Similarities and Differences in Executive Functioning Between Autistic and Non‐Autistic Adults

**DOI:** 10.1002/aur.70015

**Published:** 2025-03-13

**Authors:** Robert M. Jertberg, Sander Begeer, Hilde M. Geurts, Bhismadev Chakrabarti, Erik Van der Burg

**Affiliations:** ^1^ Section of Clinical Developmental Psychology Vrije Universiteit Amsterdam | The Netherlands and Amsterdam Public Health Research Institute Amsterdam The Netherlands; ^2^ Dutch Autism and ADHD Research Center (d'Arc), Brain & Cognition, Department of Psychology Universiteit van Amsterdam Amsterdam The Netherlands; ^3^ Leo Kannerhuis (Youz/Parnassiagroup) Amsterdam The Netherlands; ^4^ Centre for Autism, School of Psychology and Clinical Language Sciences University of Reading Reading UK; ^5^ India Autism Center Kolkata India; ^6^ Department of Psychology Ashoka University Sonipat India

**Keywords:** ADHD, attentional orienting, autism, cognitive flexibility, executive function, inhibition, working memory

## Abstract

Prior research has established differences between autistic and non‐autistic individuals across the domains of executive function (EF). While some early theories portrayed these differences as universal to the autism spectrum, recent findings have been quite mixed. Factors like small samples, the components of EF being measured, and the age and intelligence quotient (IQ) of those being compared may contribute to this diversity in results. Moreover, research suggests performance over time might fluctuate in different patterns for autistic and non‐autistic individuals. To test EF differences and the possible influence of these factors upon them, we recruited a sample of over 900 autistic and non‐autistic participants (with generally average/above average IQ levels) from 18 to 77 years of age. They completed a battery of tasks measuring inhibition, cognitive flexibility, working memory, and attentional orienting to social and nonsocial cues. We found that performance was similar between groups in our primary measures of EF, although autistic participants were consistently slower, more susceptible to the effects of spatial cueing, and more prone to certain errors in the working memory task. Differences between groups were generally not influenced by participants' age, gender, or IQ. Performance over time varied only in the working memory task. While autistic adults may still face related challenges in real life, these findings suggest that being autistic does not necessarily imply executive dysfunction on a basic cognitive level, contradicting theories assuming universal impairments therein. Moreover, the lack of influence of included demographic factors suggests that explanations for discrepancies in the literature lie elsewhere.

**Summary**

- Some research suggests that autistic individuals differ in how they switch between tasks, respond to cues, remember recently presented information, and withhold reactions.
- We tested these abilities and found that autistic people performed similarly to non‐autistic ones, albeit more slowly.
- While autistic individuals may still face some difficulties with these sorts of tasks in real life, where demands are often fast‐paced, this provides an encouraging perspective on their underlying abilities.

## 1 Introduction

Executive functions (EFs) are widely theorized to fall into three primary domains: inhibition of behavior irrelevant to objectives, cognitive flexibility (the ability to shift focus between tasks, concepts, etc.), and maintaining/manipulating mental representations in working memory (Diamond 2013; Lehto et al. 2003; Miyake et al. 2000). Each domain is essential to the ability to regulate behavior and perform goal‐oriented tasks. As such, executive *dys*function can impinge upon self‐sufficiency and quality of life. This may be especially true in autism, where difficulties with EFs have been linked to lower quality of life metrics (de Vries and Geurts 2015), symptoms of anxiety and depression (Hollocks et al. 2014; Wallace et al. 2016), and a core diagnostic criterion: restricted, repetitive behaviors and interests (Iversen and Lewis 2021; Kercood et al. 2014; South et al. 2007). In fact, some early theories posit that EF differences are central to autism and explain broader behavioral features of the condition (Hill 2004; Ozonoff and Jensen 1999; Pennington and Ozonoff 1996; Russell 1997). However, diminishing group differences over time (see Rødgaard et al. 2019, for data from a range of EF measures) and a marked heterogeneity in findings have brought these claims under increasing scrutiny, and the importance of executive dysfunction to autism remains unclear.

If EF differences are key to understanding autism, it is not only imperative to evaluate how autistic and non‐autistic individuals^1^ compare within specific domains, but also to trace the profile of differences across domains. In general, reviews and meta‐analyses have found evidence of differences between autistic and non‐autistic individuals in measures of inhibition (Geurts, van den Bergh, and Ruzzano 2014; Hlavatá et al. 2018), cognitive flexibility (Lage et al. 2024; Leung and Zakzanis 2014), and working memory (Habib et al. 2019; Kercood et al. 2014; Wang et al. 2017), although most note small sample sizes and considerable variability in outcomes. One meta‐analysis comparing the differences between autistic and non‐autistic children and adolescents across EF domains found that they are more pronounced for tasks involving flexibility and working memory than for those involving inhibition (Lai et al. 2017). Another meta‐analysis with adults over 40 years of age also found variation across domains, with significant differences between groups in working memory but not in planning or cognitive flexibility (Wang et al. 2024). In contrast, Demetriou et al. (2017) (which included children/adolescents as well as adults) found that differences across domains were comparable in magnitude, with autistic individuals performing consistently worse. While these meta‐analyses provide compelling evidence for EF differences between those with and without autism, they raise questions as to whether these differences depend on the domain in question and the type of task/dependent variables used to measure it.

For example, within the domain of inhibition, differences between groups may be more pronounced in paradigms that measure pre‐potent response inhibition (suppressing a dominant motor response) than in those that measure interference control (ignoring distractors), and they may also be more evident in reaction time (RT) data than in accuracy (Geurts, van den Bergh, and Ruzzano 2014). Differences in working memory abilities appear to increase with task complexity (Kercood et al. 2014) and are more pronounced in tasks measuring spatial rather than verbal working memory (Wang et al. 2017). For cognitive flexibility, tasks that measure perseverative errors have been found to produce the largest differences between groups (Lage et al. 2024). These findings suggest that some degree of the variability in findings is due to task demands and that behavioral differences may be more evident when experimental designs target (or combine) known problem areas.

Another task‐related factor that may influence outcomes is performance over time. There is some evidence for greater variability in RTs throughout experiments among autistic children, at least when those with co‐occurring ADHD are included in the sample (Karalunas et al. 2014). A previous study of ours showed that differences between autistic and non‐autistic individuals in the time taken to recognize emotions became smaller over the course of experiments (Jertberg et al. 2025). Relatedly, difficulties with task switching associated with autism (Lage et al. 2024; Leung and Zakzanis 2014) might be expected to translate into more pronounced differences between autistic and non‐autistic individuals at the beginning of experiments. Conversely, reported impairments in sustained attention among autistic individuals (Chien et al. 2014, 2015) might be expected to contribute to a deterioration of performance toward the end of long experiments. The nuances of these potential fluctuations in the magnitude of differences between groups over the course of experiments may be lost by the standard practice of comparing means calculated across trials. Consequently, the duration of experiments/number of trials could influence the differences between groups that emerge in these means, and variation in these factors could explain some degree of the heterogeneity in the literature. However, to date, no studies have investigated performance over time across tasks related to different EF domains in autism.

A final task‐related feature that might influence performance differences is the social nature of stimuli. Research suggests that autistic individuals show less of an attentional preference for social stimuli (Chita‐Tegmark 2016; Hedger et al. 2020), which one might expect to translate into larger differences between groups when EF tasks depend upon attending to them. However, a meta‐analysis on visual orienting found that nonsocial arrow cues produced larger differences between autistic and non‐autistic individuals than socially relevant gaze cues (Landry and Parker 2013). They also noted that most of the literature focuses on social cues and called for more research using nonsocial stimuli. As such, the importance of social factors to EF in autism deserves further investigation, which could be accomplished by comparing social and nonsocial cues within the same design.

In addition to differences between the tasks/domains in question, sampling characteristics may play an important role in the heterogeneity in findings. A study re‐evaluating several EF experiments using individual participant data found that differences in group means were driven by low‐performing subgroups rather than being characteristic of the majority of those with autism (Geurts, Sinzig, et al. 2014). This undermines theories that assume the universality of EF differences in autism and raises questions as to what exactly differentiates autistic individuals who do and do not deviate in performance. There are many factors that may be relevant to this heterogeneity (e.g., differences in the expression of autistic traits, intelligence, or language abilities). The age of the samples being compared may be particularly relevant to the extent of differences detected between them. EF abilities develop throughout childhood and adolescence before declining later in life (Best and Miller 2010; Buckner 2004). Differences between autistic and non‐autistic individuals may be more evident at certain stages in this developmental trajectory, although findings are mixed. Differences between groups were found to be smaller in adult studies than those with children in the meta‐analysis by Demetriou et al. (2017). Some studies with older adults have even reported no differences in performance on neuropsychological measures of EF between autistic and non‐autistic samples (Davids et al. 2016; Geurts et al. 2020). However, others have found objective differences in elderly (Geurts and Vissers 2012) and middle‐aged (Braden et al. 2017) samples, and while the effect size was smaller in a meta‐analysis of adults over 40 (Hedge's *g* = 0.257 vs. 0.48 in Demetriou et al. 2017, and ranging from 0.41 to 0.68 across domains in Lai et al. 2017), it was still significant (Wang et al. 2024). Furthermore, in Davids et al. (2016) and Geurts et al. (2020), the lack of objective differences in EF performance was discrepant with self‐reported difficulties in EF. These inconsistencies in outcomes warrant further research with autistic adults and underline the importance of selecting objective measures sensitive to the type of differences autistic individuals report.

Next to universality, specificity is another challenge for the EF theory of autism. ADHD is also characterized by EF differences (Barkley 1997; Willcutt et al. 2005) and has a high rate of co‐occurrence with autism (pooled current and lifetime prevalence rates of 38.5% and 40.2% among those with an autism diagnosis, according to Rong et al. 2021). Because many studies do not account for co‐occurring conditions, this complicates our understanding of the differences unique to autism. Lai et al. (2017) found that removing studies including those with co‐occurring ADHD from their meta‐analysis reduced heterogeneity and effect sizes in the inhibition and planning constructs. This suggests that the behavioral profile of autistic individuals who are also diagnosed with ADHD may differ from those who are not. One might expect more pronounced EF difficulties at their intersection, given that both conditions are associated with them. However, symptoms of ADHD are often treated with medications that may alleviate EF difficulties (Hai et al. 2022; Kempton et al. 1999). Since those with both autism and ADHD take these medications at considerably higher rates than those with autism alone (Frazier et al. 2011), this could lead to similar or even higher performance among autistic individuals who also have an ADHD diagnosis. Existing EF studies focusing on both autism and ADHD included mainly children (Antshel et al. 2016; Craig et al. 2016; Gargaro et al. 2011; Taurines et al. 2012), and only a few adult studies have investigated the potential influence of concurrent ADHD and autism on EF differences. For example, one pre‐potent response experiment found that ADHD symptoms were not correlated with any outcome measures besides response variability in an autism group; however, the authors noted that their sample only included six individuals with an ADHD diagnosis (Torenvliet et al. 2023). As such, further research is necessary to understand the intimate relationship between autism and ADHD and how it might influence EF.

Pinpointing EF differences that may exist in autism is not only crucial to our understanding of the condition and theories that revolve around their significance to it. It may also allow more effective therapeutic interventions, given that cognitive training (particularly strategy‐based cognitive training) has shown some promise in ameliorating EF difficulties among autistic children who face them. However, it should be noted that there are still questions about the generalizability of these benefits and whether they hold for adults (Cavalli et al. 2022; de Vries et al. 2021; Kaur et al. 2024; Pasqualotto et al. 2021). Small samples, methodological inconsistency, and lack of variation in relevant demographic factors have all impeded this endeavor.

To address the resulting gaps in the literature, we have conducted a study incorporating a large sample with a wide age range of autistic and non‐autistic adults, a battery of tasks tapping into each of the primary areas of EF, and novel analytic strategies to chart performance over the course of the experiments. Tasks were carefully selected to target potential problem areas, such as pre‐potent response inhibition, complex spatial working memory demands, switching between related tasks, and social versus nonsocial stimuli. This has allowed us to ask: (a) whether autistic adults differ in measures of inhibition, cognitive flexibility, attentional allocation, and/or working memory, (b) what demographic factors (particularly age and co‐occurring ADHD) influence these potential differences, and (c) how performance varies over time in both groups. Given the literature, we predicted that autistic participants would show impairments in each of the four measures of EF.

## 2 Methods

*Participants*: The four experiments described in this article were part of a larger battery of two 45‐min sessions (see Jertberg et al. 2024, 2025), presented in the same fixed order for both groups. A total of 708 autistic participants were recruited via the Netherlands Autism Register (NAR, https://nar.vu.nl/, a cohort of Dutch autistic and non‐autistic volunteers), and 533 non‐autistic participants were recruited via Prolific Academic (an online participant recruitment platform; see www.prolific.com) as well as the NAR. NAR and Prolific Academic participants were compensated with a gift card (€15) or paid £15, respectively. Autistic participants reported having a formal diagnosis by a registered clinician; non‐autistic participants reported no autism diagnosis. To join the NAR, non‐autistic participants must also confirm that they do not have any immediate relatives with an autism diagnosis. All participants were fluent in Dutch and provided informed consent. The experiments were approved by the ethical committee from the Vrije Universiteit Amsterdam (VCWE‐2020‐041R1) in accordance with the Netherlands Code of Conduct for Research Integrity and the revised declaration of Helsinki. All experiments were programmed and conducted online using Neurotask (www.neurotask.com). Exclusion rates differed according to demographic data availability and the criteria specified in our pre‐registration (As Predicted # 160633, https://aspredicted.org/J61_Z9R).

Note that in addition to the pre‐registered exclusion criteria, for the go/no‐go task, we removed 21 participants who made more than 20% errors (their mean error rate was 69.6%, compared to 1.3% for the whole group). In the trail making task (TMT), the last response was not recorded due to a programming issue. Consequently, we excluded 11 participants from further analyses, as their last response recorded was an erroneous one (making it impossible to derive the correct RT). Finally, six participants were excluded from the chessboard task because they never responded correctly. Detailed information on exclusion criteria can be found in Figures S1–S4. Table 1 depicts the demographic information for the participants included in each experiment. Experiments 1 and 3 have considerably lower numbers of participants due to being positioned in the second half of the battery, which was completed in a separate sitting and suffered some attrition as a result. Participants were included in the analyses for each task they completed and excluded from those they did not.

**TABLE 1:** Demographic information for each experiment.

	Experiment 1: go/no‐go		Experiment 2: trail making		Experiment 3: chessboard		Experiment 4: arrow/gaze	
	Autism (*N* = 374)	No autism (*N* = 353)	Autism (*N* = 564)	No autism (*N* = 423)	Autism (*N* = 396)	No autism (*N* = 385)	Autism (*N* = 557)	No autism (*N* = 416)
Gender*								
Women (%)	67.9	49.2	64.4	51.1	66.7	48.8	64.4	51
Men (%)	32.1	50.8	35.6	48.9	33.3	51.2	35.6	49
Age*	44.9 (14.2)	31.9 (11.9)	44.6 (14.0)	33.2 (13.0)	44.9 (14.1)	32.1 (12.2)	44.5 (13.9)	32.8 (12.9)
Age range	18–77	18–77	18–77	18–76	18–77	18–74	18–77	18–74
AQ‐28*	83.0 (10.6)	60.5 (11.1)	83.0 (10.8)	59.9 (11.3)	83.3 (10.8)	60.6 (11.1)	82.9 (10.8)	59.9 (11.3)
AQ‐28 range	46–109	30–96	46–109	30–96	46–109	30–96	46–109	30–96
ICAR^a^	0.53 (0.21)	0.51 (0.19)	0.53 (0.21)	0.52 (0.19)	0.53 (0.21)	0.52 (0.19)	0.53 (0.21)	0.52 (0.19)
ICAR range	0–0.94	0.0–0.94	0–0.94	0.06–0.94	0–0.94	0–0.94	0–0.94	0–0.94

*Note*: Standard deviations are shown in parentheses. Asterisks signify significant group differences (*p* < 0.05).

Abbreviations: AQ: Autism Quotient; ICAR: International Cognitive Ability Resource (abbreviated intelligence quotient test).

^a^ The ICAR project is an open resource for online cognitive tasks. We used the ICAR‐16 intelligence test, which correlates strongly with full‐scale IQ (Young and Keith 2020) and has been shown to be age and sex invariant (Young et al. 2019). We report proportions of correct responses, which we use as covariates in subsequent analyses.

### 2.1 Experiment 1: Inhibition (Go/No‐Go Task)

**FIGURE 1:**
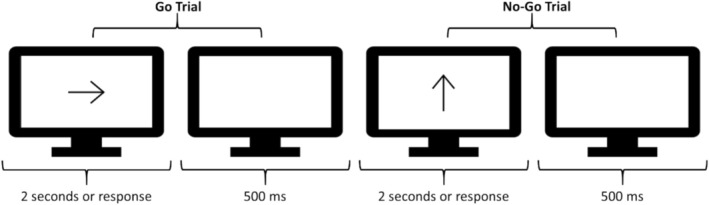
Example trial sequence from the go/no‐go task. A trial started with the presentation of an arrow. Participants were instructed to press the z‐ or m‐key if the arrow was facing to the left or right, respectively (i.e., a Go trial). Participants were instructed to withhold their response if the arrow was facing up (i.e., a No‐Go trial). The monitor provides a proportionate representation of the space the stimuli took up in the web browser.

*Stimuli and Procedure*: Figure 1 depicts a sequence of trials in the Go/No‐Go task, inspired by the classic paradigm originally developed by Donders (1969). This task (and those to follow) was chosen because it has been used extensively to study the underlying domains in question in both neurotypical and neurodivergent groups.

On every trial, a black arrow (with a length equal to 50% of available screen space within the browser) was displayed at the center of the screen. Participants were instructed to press the z‐ or m‐key as quickly as possible if the arrow was facing to the left or right, respectively (i.e., a Go trial) or to withhold their response if the arrow was facing upwards (i.e., No‐Go trial). The arrow was shown until participants responded or 2 s elapsed. Subsequently, a white screen was shown for 500 ms, after which the next trial was initiated. Participants completed 10 practice Go trials and 4 No‐Go trials, followed by 220 experimental Go trials (half right, half left) and 80 No‐Go trials. Stimulus presentation was randomized. For this task (and the following), written instructions were displayed on the screen prior to the beginning of the experiment.

### 2.2 Experiment 2: Cognitive Flexibility (TMT)

**FIGURE 2:**
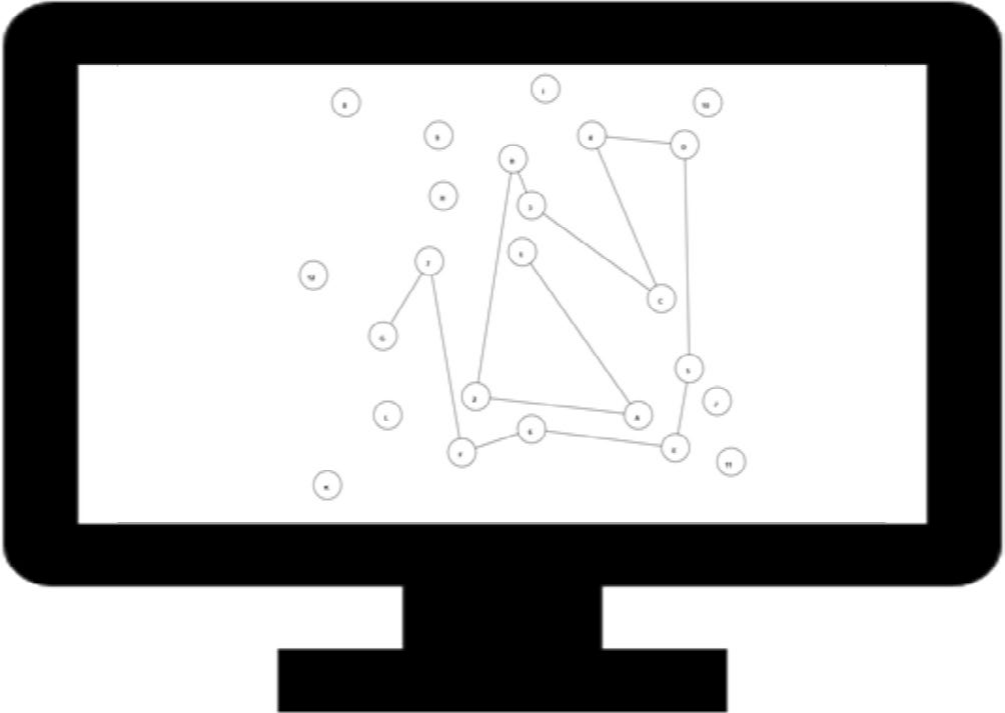
Illustration of the trail making task setup. In the numeric condition, participants were instructed to click on the nodes in numerical order. In the alphanumeric condition (shown in this illustration), the participants were instructed to alternate between numbers and letters (e.g., 1‐A‐2‐B‐3‐C …). In both conditions, when participants clicked the correct node, a line would appear connecting it with the previous one. The monitor provides a proportionate representation of the space the stimuli took up in the web browser.

*Stimuli and Procedure*: The TMT, a classic neuropsychological test developed by Reitan (1955), consisted of two separate conditions. In part A (the numeric condition), the display consisted of small circles (radii = 3% of available screen space within the browser) numbered 1–25, and participants were instructed to respond by clicking through the circles as quickly and accurately as possible in numerical order. In part B (the alphanumeric condition), the display consisted of 24 circles with letters (A–L) and numbers (1–12), and participants were instructed to alternate between them in the correct order (e.g., 1‐A‐2‐B‐3‐C…). On both parts, hovering above a node caused it to turn a light shade of gray. When participants clicked the correct node, it would flash a darker shade of gray for 500 ms, and a line would appear connecting it with the previous node. When they clicked an incorrect node, it would flash red for 500 ms. Figure 2 depicts an illustration of the alphanumeric condition (completed partially). Participants first completed the numeric condition, followed by the alphanumeric condition, without practice.

### 2.3 Experiment 3: Working Memory (Chessboard Task)

**FIGURE 3:**
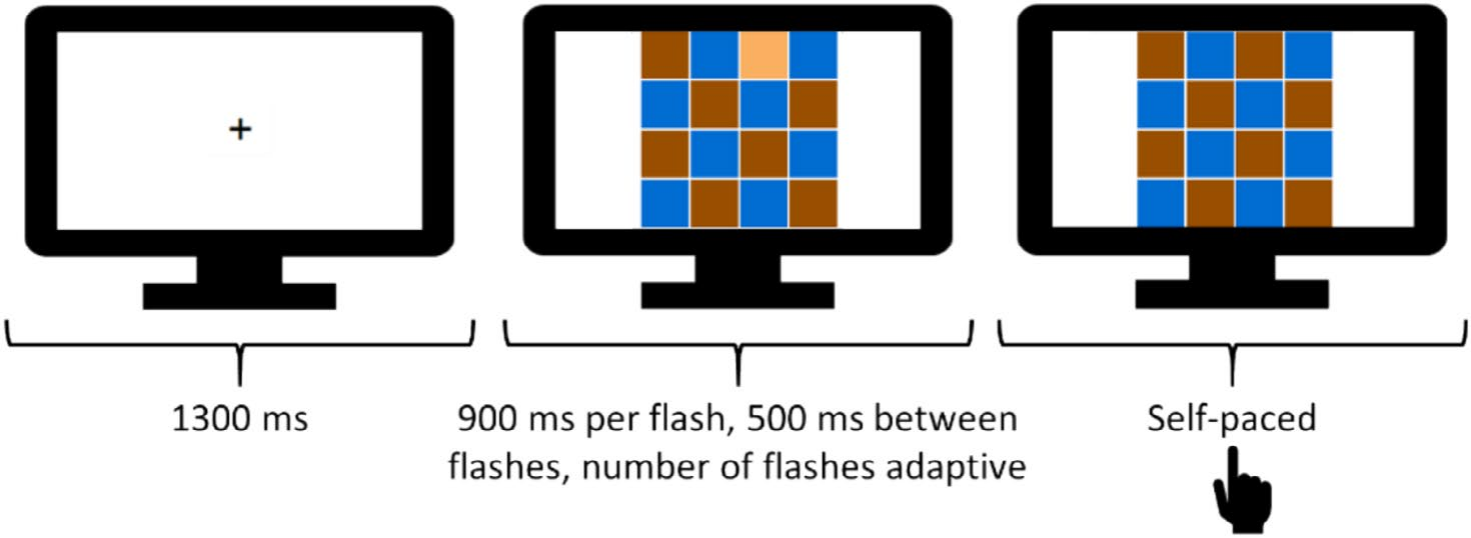
Example trial sequence from the chessboard task. A number of squares (from three up, according to performance) flashed sequentially, always with at least one blue square flashing before an orange one. Participants then responded by clicking all the orange and then all the blue squares in the order in which they flashed. The monitor provides a proportionate representation of the space the stimuli took up in the web browser.

*Stimuli and Procedure*: Figure 3 illustrates an example trial sequence. This task is an adaptation of that used in Dovis et al. (2014).

A trial started with a black fixation cross in the center of a white screen for 1300 ms. Subsequently, a 4 × 4 grid of alternating orange and blue squares (selected to be color blind friendly, with heights/widths of 24% of available screen space within the browser) separated by thin white borders (1% of available screen space) appeared on the screen. Then, several squares flashed sequentially in lighter versions of their respective colors for 900 ms each (the interval between two flashes was 500 ms). Each onset was accompanied by a “ding” sound. The squares that flashed were randomly selected, with the constraints that at least one of each color flashed and that at least one blue square flashed before the last orange one. Participants were instructed to respond by clicking all the orange squares and then all the blue squares, both in the order in which they lit up. The same “ding” sound was played when each square was clicked. The next trial began as soon as the participant clicked as many squares as had flashed (irrespective of whether they were the same ones). The task difficulty (i.e., the number of flashing squares) was adaptive, beginning with a span of three squares flashing. After two consecutive correct/incorrect trials, the span would increase/decrease by one square. Note that the minimal span was set to three. Participants completed five practice trials, receiving feedback on the type of any errors they made (clicking the wrong orange square, the wrong blue square, or a blue square before an orange square) after each. The span then reset to three (if it had changed), and they completed 30 experimental trials without feedback.

### 2.4 Experiment 4: Social and Nonsocial Attentional Orientation (Arrow/Gaze Cueing Task)

**FIGURE 4:**
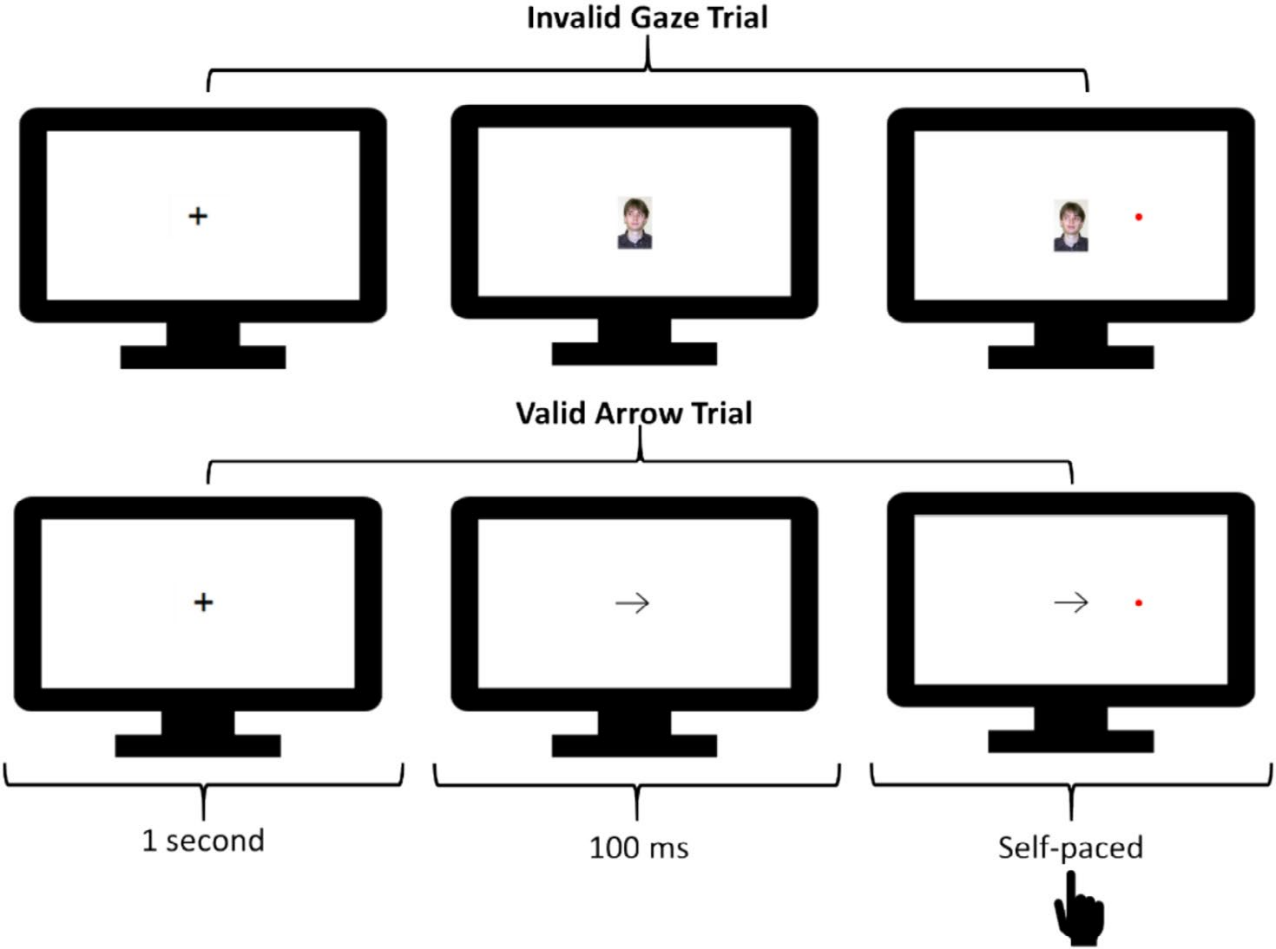
Sequence of trials from the arrow/gaze cueing task. Trials started with the presentation of a fixation cross, then an arrow or gaze cue (which was valid 50% of the time, i.e., spatially uninformative), and then a red circle (the target) on either the right or the left of the cue. Participants responded to the location of the target by pressing the z‐key for those on the left and the m‐key for those on the right. The monitor provides a proportionate representation of the space the stimuli took up in the web browser.

*Stimuli and Procedure*: Figure 4 provides an example sequence of trials. This task was inspired by Driver et al. (1999).

Trials began with a black fixation cross for 1 s. Next, either a left‐ or right‐facing arrow or gaze cue (gaze stimuli taken from Bayliss et al. 2006) was shown. Cues were equivalent in width (20% of available screen space within the browser) and centered so that either the eyes or point of the arrow were aligned with the point at which targets appeared on the vertical axis. The target, a red circle with a radius of 2% of the available screen space within the browser, then appeared 100 ms after the cue onset to either the left or right of the screen. The cue pointed in the direction in which the target would appear half of the time. Participants were instructed to respond as quickly and accurately as possible to the location of the target by pressing the z‐key if it appeared on the left and the m‐key if it appeared on the right. The next trial was initiated when participants made a response. Participants completed 8 practice trials before 80 experimental trials in which the validity and type of the cue (as well as the location of the target) were evenly balanced and randomly intermixed.

## 3 Results

Alpha was set to 0.05, but Bonferroni corrected for multiple comparisons according to the number of dependent variables measured for each experiment. Statistical tests were conducted using Just Another Statistical Program (JASP, version 0.17.3.0, see Love et al. 2019) and Jamovi (version 2.3.28, see The Jamovi Project 2024). For each experiment, statistics are reported in the main manuscript for the primary measure(s) of the relevant construct and performance over time analysis, and any other significant group effects are stated at the end of the section. Statistics for all other dependent variables can be found in the Supporting Information (along with follow‐up Bayesian analyses on the primary dependent variables of interest). Results are reported starting with RT and progressing to error rates for consistency.

### 3.1 Experiment 1: Inhibition (Go/No‐Go Task)

**FIGURE 5:**
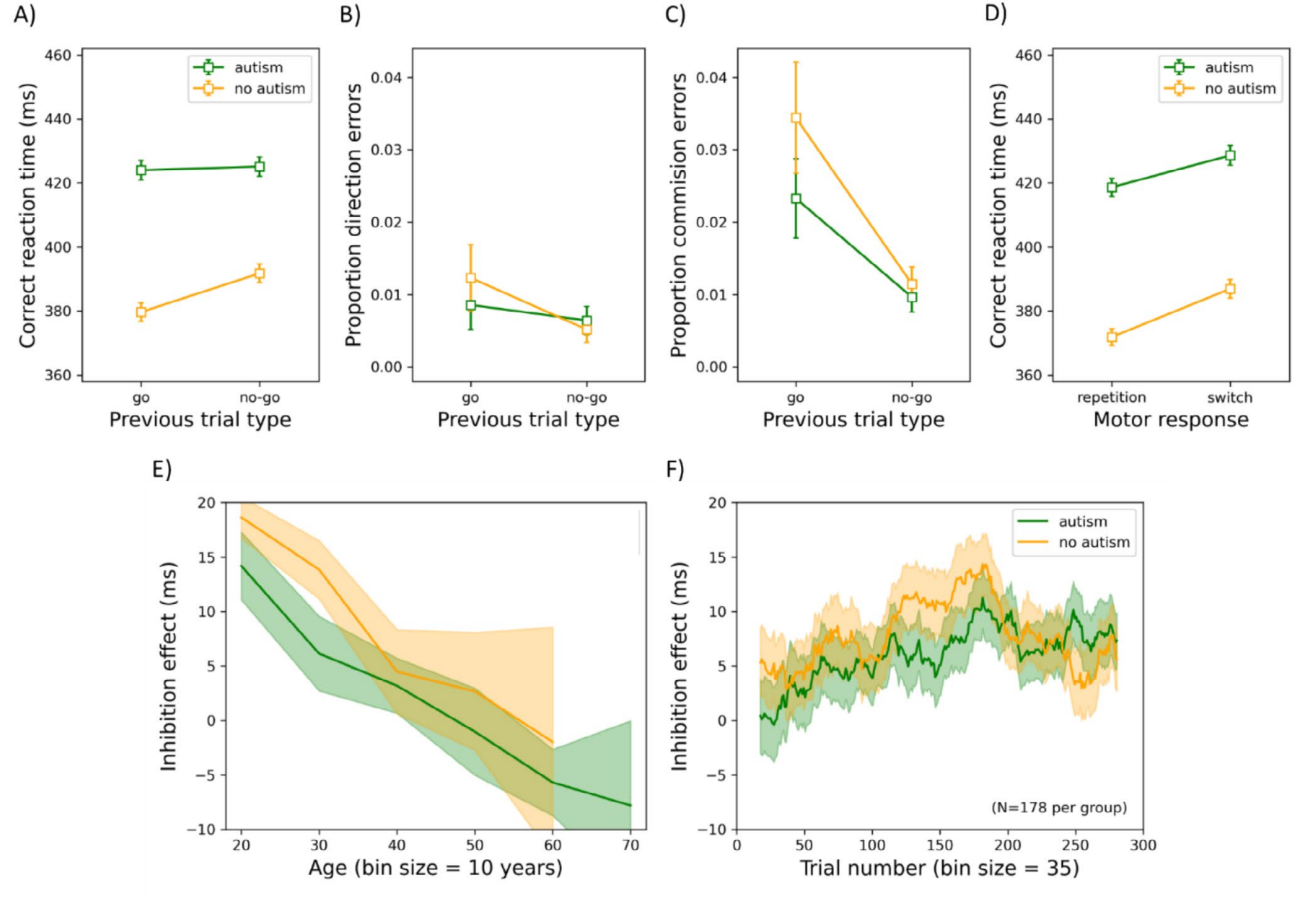
Results of the Go/No‐Go task. Mean correct reaction time (panel A), mean proportion of directional errors (panel B), and mean proportion of commission errors (panel C) as functions of the previous trial type for participants with or without autism. Panel (D) illustrates the mean correct reaction time as a function of motor response (i.e., repetition trials versus switch trials) for people with and without autism. Note that the current and previous trials were both correct Go trials. Panel (E) illustrates the inhibition effect (RT after a previous no‐go trial—RT after a previous go trial) as a function of age (divided into bins of 10 years). Note that bins with fewer than 10 participants were not shown. Panel (F) depicts the inhibition effect as a function of trial number (divided into bins of 35 trials). In other words, the first bin represents the mean inhibition effect for trials 1–35, the second bin represents the mean over trials 2–36, and so forth (see also Van der Burg et al. 2015). Note that for panel (F), age and gender were individually matched (*N* = 178). In all panels, the error bars represent the standard error of the mean.

Practice trials and the first experimental trial were excluded from further analyses. Trials on which participants responded too slowly (1.4% of the trials; RT was > mean RT + 3SD) or too quickly (0.4% of the trials; RT was < mean RT − 3SD) were also removed, as per our pre‐registration. Finally, another 0.04% of the trials were excluded from further analyses as participants opened another application (e.g., email, Facebook). In Figure 5A–C, the mean correct RT, the mean proportion of directional errors, and the mean proportion of commission errors (responses on no‐go trials) are shown as functions of the previous trial type (Go or No‐Go) for participants with or without autism. Panel D depicts the mean correct RT for autistic and non‐autistic individuals on trials following correct Go trials, as a function of whether the target was on the same side (repetition) or the opposite side (switch). Additionally, the inhibition RT effect (derived by subtracting RTs from trials following Go trials from those of trials following No‐Go trials) is plotted as a function of age and trial number (panel E–F).

For each dependent variable, we conducted a mixed‐measures analysis of variance (ANOVA) with previous trial type as a repeated‐measures factor, ICAR score and age as covariates, and group and gender as between‐subjects factors. In this and following experiments, we chose to include gender/age as a cofactor/covariate (as is conventional in the field) due to evidence of their influences on cognitive performance, which may otherwise have obscured insight into differences related to autism (Levine et al. 2021; Maylor et al. 2007; McCarrey et al. 2016). While the literature on differences between men and women generally is quite mixed, there is some evidence of cognitive differences between autistic men and women more specifically (Lai et al. 2012), and the inclusion of gender as a cofactor is recommended when studying neurodevelopmental conditions (Bölte et al. 2023). We included ICAR scores because research comparing autistic/non‐autistic individuals often matches or co‐varies for IQ (e.g., Liss et al. 2001; Sucksmith et al. 2013). However, due to concerns about doing so in studies of EF and when comparing differing neurodevelopmental populations (Dennis et al. 2009), we conducted sensitivity analyses excluding ICAR scores as covariates, the results of which can be found in the Overarching Analyses section. As we analyzed three dependent variables, alpha was set to 0.017.

*Mean Correct RT*: (Note: commission error rates are also frequently reported as the primary metric of inhibition; relevant analyses can be found in the Supporting Information) The ANOVA yielded a main effect of group (*F*(1, 721) = 7.042, *p* = 0.008, *η*_p_^2^ = 0.010), such that autistic participants (mean averaged across previous trial types: 425 ms) were generally slower than non‐autistic ones (386 ms). Age was positively related to RT (i.e., higher age, slower response; *F*(1, 721) = 267.679, *p* < 0.001, *η*_p_^2^ = 0.271); ICAR score was negatively related (i.e., higher IQ, faster response, *F*(1, 721) = 13.565, *p* < 0.001, *η*_p_^2^ = 0.018). Previous trial type also had a significant main effect (*F*(1, 721) = 25.051, *p* < 0.001, *η*_p_^2^ = 0.034), with participants responding more slowly on trials following No‐go trials (408 ms) than Go trials (402 ms). There was a significant interaction between previous trial type and age (*F*(1, 721) = 34.178, *p* < 0.001, *η*_p_^2^ = 0.045), with age correlating negatively with the inhibition RT effect (Pearson *r* = −0.285, *p* < 0.001), as seen in Figure 5d. The interaction between group and previous trial type did not survive Bonferroni correction (*F*(1, 721) = 4.207, *p* = 0.041, *η*_p_^2^ = 0.006). All other *F* ≤ 2.995; all other *p* ≥ 0.084.

*Time‐Series Analysis* ^2^: To investigate potential changes in performance over time in the experiment, we conducted a follow‐up analysis with groups (*N* = 178 per group) individually matched on age and gender, which were confirmed to still differ significantly in AQ but not in ICAR scores. (Note: this matching procedure applies to all subsequent time‐series analyses.) We divided the trials into bins of 35 trials and calculated the magnitude of the inhibition effect (the difference in RT between trials following *N*o‐*G*o vs. *G*o trials) following a walking average (see also Van der Burg et al. 2015 for a similar approach). In other words, the first bin corresponds to the mean inhibition RT effect over trial 1–35, the second bin over trial 2–36, and so forth. The bin size was chosen to allow calculation of the inhibition effect for each participant after accounting for lost/erroneous trials. We conducted a repeated‐measures ANOVA on the inhibition RT effect with trial bin as a repeated‐measures factor and group as a between‐subjects variable. Group had no significant main effect (*F*(1, 354) = 0.340, *p* = 0.560, *η*_p_^2^ = 9.602 × 10^−4^). While bin had a significant main effect (*F*(263, 93,102) = 2.810, *p* < 0.001, *η*_p_^2^ = 0.008), with the potency of the inhibition effect increasing over the course of trials, it did not interact with group (*F*(263, 93,102) = 0.978, *p* = 0.588, *η*_p_^2^ = 0.003).

*Significant group × previous trial type interaction on directional error rates*: Previous trial type interacted with group (*F*(1, 721) = 7.407, *p* = 0.007, *η*_p_^2^ = 0.010), such that autistic individuals exhibited a smaller difference in directional error rates between previous trial types (0.2%) than non‐autistic ones (0.7%), *t*(725) = −4.579, *p* < 0.001.

### 3.2 Experiment 2: Cognitive Flexibility (TMT)

**FIGURE 6:**
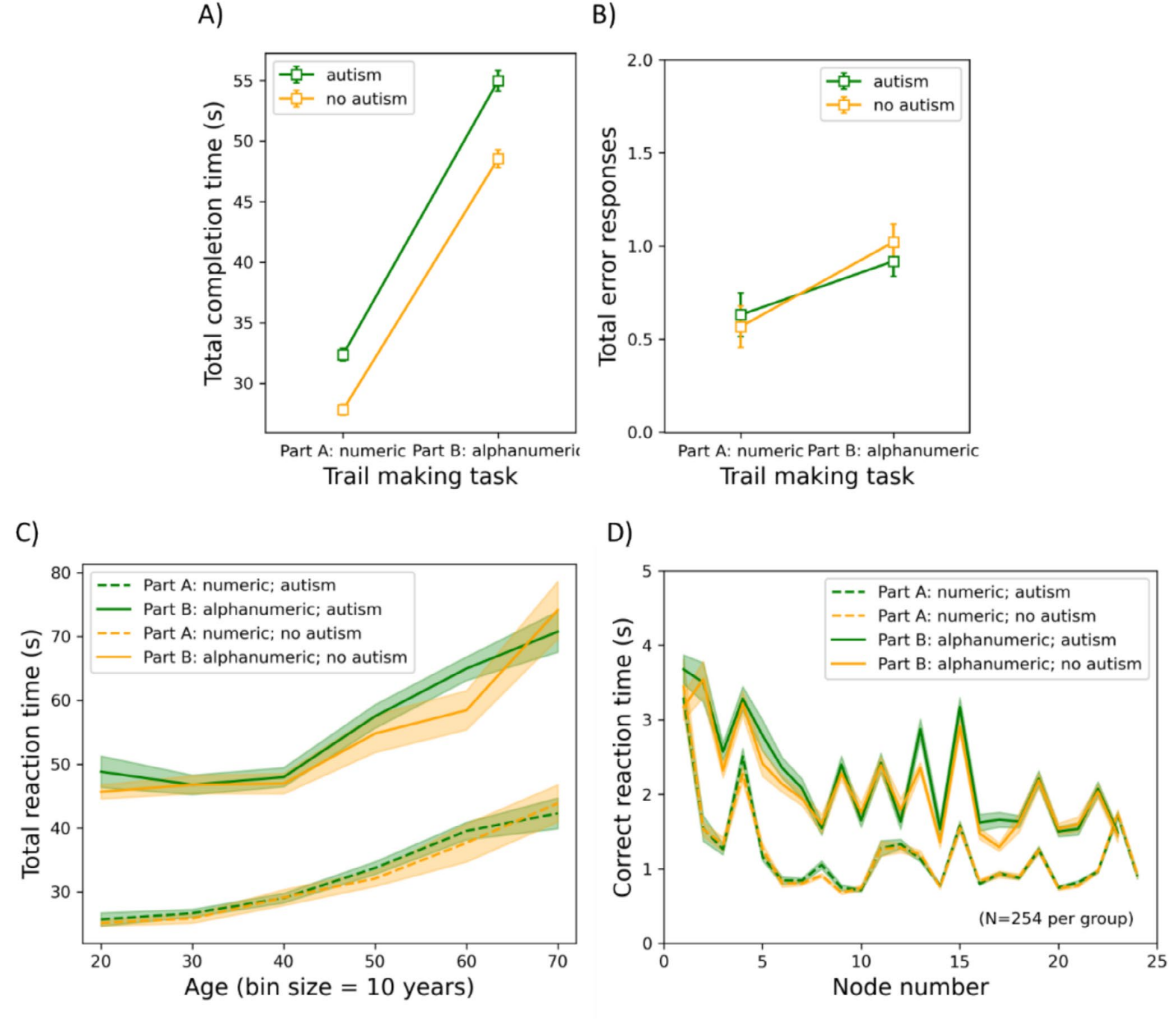
Results of the trail making task. Mean total completion time (panel A) and mean number of errors (panel B) as functions of task part (i.e., A: Numeric versus B: Alphanumeric) for participants with or without autism. Panel (C) illustrates the mean total completion time as a function of age (divided into bins of 10 years). Note that bins with fewer than 10 participants were not shown. Panel (D) depicts the mean correct reaction time as a function of node. Note that for panel (D), age and gender were individually matched (*N* = 178). In all panels, the error bars represent the standard error of the mean.

The results of the TMT are shown in Figure 6. Here, the mean total completion time and the number of errors are shown as functions of the TMT part (part A and B) for participants with and without autism (panels A‐B). Additionally, the mean total completion time is plotted as a function of age (panel C), and the mean RT is shown per node (panel D). For each dependent variable, we conducted a mixed‐measures ANOVA with TMT part as a repeated‐measures factor, ICAR score and age as covariates, and group and gender as between‐subjects factors. As there were two dependent variables, alpha was set to 0.025.

*Total completion time*: The group did not have a significant main effect (*F*(1, 981) = 3.532, *p* = 0.060, *η*_p_^2^ = 0.004), nor did it interact with TMT part (*F*(1, 981) = 2.441, *p* = 0.119, *η*_p_^2^ = 0.002). Age had a positive relationship with total completion time (*F*(1, 981) = 252.128, *p* < 0.001, *η*_p_^2^ = 0.204), whereas ICAR score had a negative relationship (*F*(1, 981) = 136.078, *p* < 0.001, *η*_p_^2^ = 0.122). Gender also had a significant main effect (*F*(1, 981) = 14.255, *p* < 0.001, *η*_p_^2^ = 0.014), with men having a longer mean completion time across parts (87 s) than women (80 s). Part had a significant main effect (*F*(1, 981) = 203.479, *p* < 0.001, *η*_p_^2^ = 0.172), with the alphanumeric part of the TMT (i.e., part B; 52 s) taking longer than the numeric part (part A; 30 s) to complete. Part also interacted positively with age (*F*(1, 981) = 8.903, *p* = 0.003, *η*_p_^2^ = 0.009), gender (*F*(1, 981) = 27.096, *p* < 0.001, *η*_p_^2^ = 0.027), and ICAR (*F*(1, 981) = 60.693, *p* < 0.001, *η*_p_^2^ = 0.058). The difference in completion times between parts correlated positively with age (*r* = 0.132, *p* < 0.001) and negatively with ICAR score (*r* = −0.244, *p* < 0.001). The difference was larger for men (24.898 s) than for women (19.611 s) (*t*(985) = 5.372, *p* < 0.001). All other *F* ≤ 0.426; all other *p* ≥ 0.514.

*Time‐series analysis*: With an age‐ and gender‐matched subgroup (*N* = 254 per group), we conducted a repeated‐measures ANOVA on the mean RT with node as a within‐subjects factor and group as a between‐subjects factor for each part of the task (respectively). Due to violation of the assumption of sphericity, Huynh‐Feldt corrections were applied. There was no main effect of group for either part (A: *F*(1, 506) = 0.377, *p* = 0.540, *η*_p_^2^ = 7.44 × 10^−4^; B: *F*(1, 506) = 3.360, *p* = 0.067, *η*_p_^2^ = 0.007), nor did it interact with node number (A: *F*(23, 11,638) = 0.416, *p* = 0.718, *η*_p_^2^ = 8.215 × 10^−4^; B: *F*(22, 11,132) = 1.171, *p* = 0.288, *η*_p_^2^ = 0.002); however, there were main effects of node number for both parts (A: *F*(23, 11,638) = 121.021, *p* < 0.001, *η*_p_^2^ = 0.193; B: *F*(22, 11,132) = 57.497, *p* < 0.001, *η*_p_^2^ = 0.102).

### 3.3 Experiment 3: Working Memory (Chessboard Task)

**FIGURE 7:**
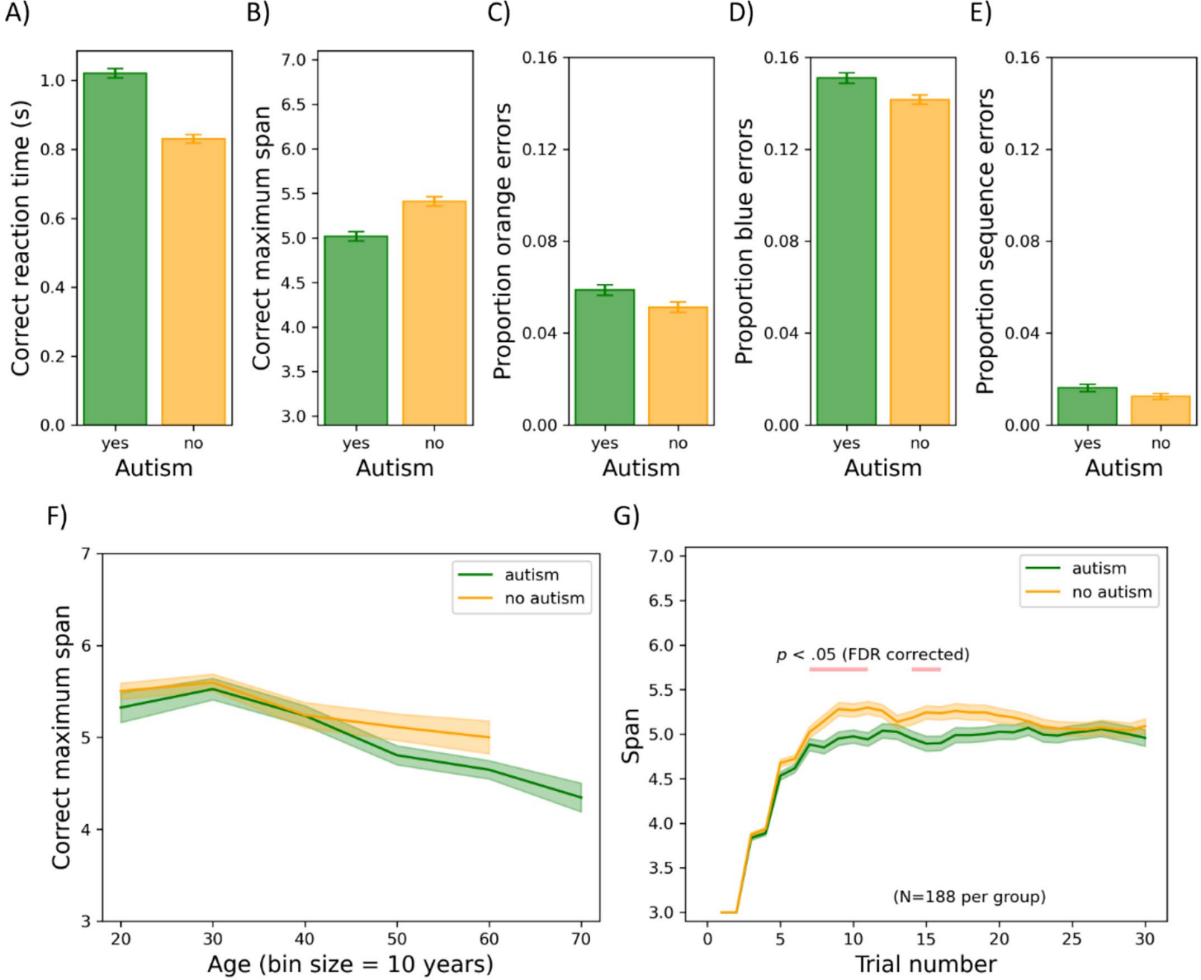
Results of the chessboard task. Mean correct total reaction time divided by current span (panel A), mean correct maximum memory span (the highest number of blocks clicked twice consecutively) (panel B), and the proportion of orange, blue, and sequence errors divided by the current span (panels C–E) are shown for participants with or without autism. Panel (F) illustrates the mean correct maximum span as a function of age (divided into bins of 10 years). Note that bins with fewer than 10 participants were not shown. Panel (G) depicts the average current span as a function of trial number. Note that for panel (G), age and gender were individually matched (*N* = 191). In panel (G), significant group differences per bin (*p* < 0.05, false discovery rate corrected) are indicated by the pink bar. In all panels, the error bars represent the standard error of the mean.

Practice trials were excluded from further analyses. Furthermore, RT outliers on which participants responded too slowly (1.14%; RT was > mean RT + 3SD) or too quickly (0.0%; RT was < mean RT − 3SD) were excluded from further analyses for calculating the mean RT. The results of the chessboard task are shown in Figure 7. Here, the mean correct RT, mean correct maximum memory span (the highest number of blocks clicked twice consecutively), and orange, blue, and sequence error rates are shown for both groups (panels A–E). Orange/blue errors refer to errors in which participants press a block of the corresponding color in the wrong order. Sequence errors refer to errors in which participants press a blue block before the last orange block. Error rates and RT were calculated by dividing their given value on each trial by the span at the beginning of that trial, then averaging across these quotients. Additionally, the mean correct span is plotted as a function of age and trial number (panels F–G). For each dependent variable, we conducted an analysis of covariance (ANCOVA) with ICAR score and age as covariates and group and gender as between‐subjects factors. As there were five dependent variables, alpha was set to 0.010.

*Mean correct RT*: There was a significant main effect of group (*F*(1, 769) = 23.853, *p* < 0.001, *η*_p_^2^ = 0.030), such that autistic participants (1021 ms) were slower on average than non‐autistic participants (831 ms). There was also a significant main effect of gender (*F*(1, 769) = 14.248, *p* < 0.001, *η*_p_^2^ = 0.018), such that women (962 ms) were slower on average than men (880 ms), but gender also interacted with group (*F*(1, 769) = 11.088, *p* < 0.001, *η*_p_^2^ = 0.014). Autistic women (1000 ms) were faster (*t*(391) = 2.114, *p* < 0.035) than autistic men (1062 ms); conversely, non‐autistic men (759 ms) were faster (*t*(380) = 6.175, *p* < 0.001) than non‐autistic women (907 ms). Age correlated positively with mean correct RT (*F*(1, 769) = 116.064, *p* < 0.001, *η*_p_^2^ = 0.131). The effect of ICAR score (*F*(1, 769) = 5.695, *p* = 0.017, *η*_p_^2^ = 0.007) did not survive Bonferroni correction.

*Mean correct maximum span*: The main effect of group did not survive Bonferroni correction (*F*(1, 769) = 5.952, *p* = 0.015, *η*_p_^2^ = 0.008). Age had a negative relationship with span (*F*(1, 769) = 60.938, *p* < 0.001, *η*_p_^2^ = 0.073), whereas ICAR score had a positive relationship (*F*(1, 769) = 62.503, *p* < 0.001, *η*_p_^2^ = 0.075). All other *F* ≤ 6.049; all other *p* ≥ 0.014.

*Time‐series analysis*: To evaluate performance over time, we created two age‐ and gender‐matched subgroups (*N* = 188 per group) as in the previous analyses and conducted a repeated‐measures ANOVA on the current span with each trial as a repeated‐measures level and group as a between‐subjects factor. Note that for this analysis, we did not exclude trials in which participants were too slow/fast (1.14% of trials), because it would lead to empty cells in the repeated‐measures design. Group did not have a significant main effect on current span (*F*(1, 374) = 4.883, *p* = 0.028, *η*_p_^2^ = 0.013). Trial number did have a significant main effect (*F*(29, 10,846) = 238.262, *p* < 0.001, *η*_p_^2^ = 0.389), with span increasing over the course of trials before appearing to plateau. It also interacted with group (*F*(29, 10,846) = 2.355, *p* < 0.001, *η*_p_^2^ = 0.006). For six trials towards the middle of the task, the non‐autistic group seemed to outperform the autistic group, while at the start and end of the tasks, no differences emerged. Figure 7g shows the trials for which the groups differed in span (according to *t*‐tests with FDR corrections for multiple comparisons) in pink.

*Significant group main effect on orange error rates*: There was a significant main effect of group on the rate of orange errors (*F*(1, 769) = 9.766, *p* = 0.002, *η*_p_^2^ = 0.013), such that autistic participants made more orange errors (0.059) than non‐autistic participants (0.051).

### 3.4 Experiment 4: Social and Nonsocial Attentional Orientation (Arrow/Gaze Cueing Task)

**FIGURE 8:**
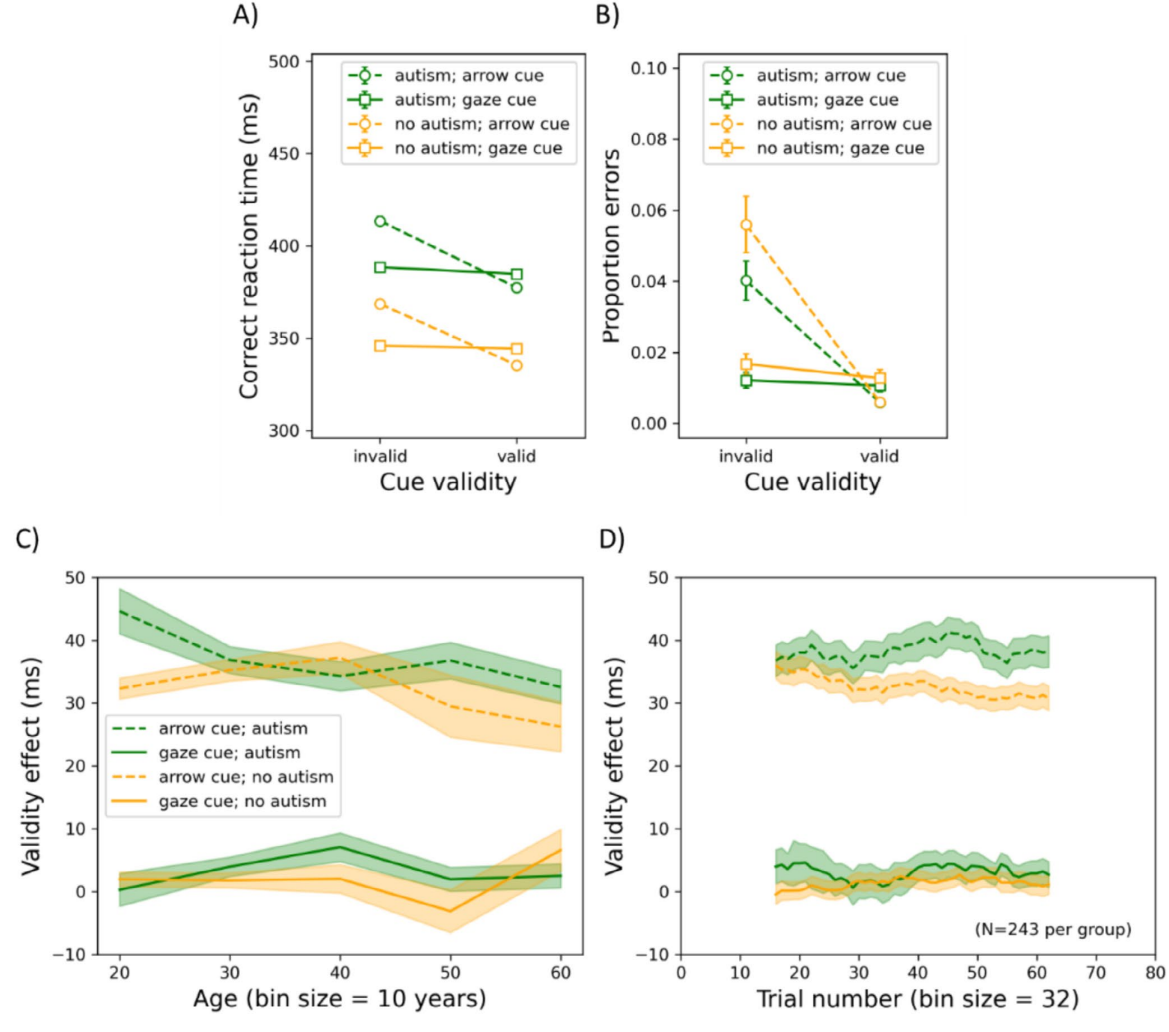
Results of the arrow/gaze cueing experiment. Mean correct reaction time (panel A) and mean proportion of errors as functions of cue validity and cue type (arrow or gaze) for participants with or without autism. Panel (C) illustrates the cueing validity effect (RT after an invalid cue—RT after a valid cue) as a function of age (divided into bins of 10 years) for both groups and cue types. Note that bins with fewer than 10 participants were not shown. Panel (D) depicts the cueing validity effect as a function of trial number (divided into bins of 32 trials) for both groups and cue types. In other words, the first bin represents the mean cue validity effect for trials 1–32, the second bin represents the mean over trials 2–33, and so forth. Note that for panel (E), age and gender were individually matched (*N* = 243). In all panels, the error bars represent the standard error of the mean.

Practice trials were excluded from further analyses. Furthermore, RT outliers on which participants responded too slowly (1.4% of the trials; RT was > mean RT + 3SD) or too quickly (0.2% of the trials; RT was < mean RT − 3SD) were excluded from further analyses. Finally, another 0.03% of the trials were excluded from further analyses as participants opened another application. The results of the arrow/gaze cueing experiment are shown in Figure 8. Here, the mean correct RT and the mean proportion of errors are shown as functions of cue validity and cue type (arrow or gaze) for participants with or without autism. Additionally, the effect of cue validity is plotted as a function of age and trial number (panels C and D). For each dependent variable, we conducted a mixed‐measures ANOVA with cue type and validity as repeated‐measures factors, ICAR score and age as covariates, and group and gender as between‐subjects factors. As there were two dependent variables, alpha was set to 0.025.

*RT*: The group had a significant main effect (*F*(1, 967) = 23.391, *p* < 0.001, *η*_p_^2^ = 0.024), such that autistic participants (391 ms) were slower than non‐autistic ones (349 ms). There was also a main effect of gender (*F*(1, 967) = 5.876, *p* = 0.016, *η*_p_^2^ = 0.006), such that women (377 ms) were slower than men (368 ms). Age was positively related to RT (*F*(1, 967) = 249.359, *p* < 0.001, *η*_p_^2^ = 0.205); ICAR score was negatively related (*F*(1, 967) = 24.103, *p* < 0.001, *η*_p_^2^ = 0.024). Cue type had a main effect (*F*(1, 967) = 36.610, *p* < 0.001, *η*_p_^2^ = 0.036), such that participants were slower overall following arrow (377 ms) than gaze (369 ms) cues. Cue validity also had a main effect (*F*(1, 967) = 111.596, *p* < 0.001, *η*_p_^2^ = 0.103), with participants being slower following invalid (382 ms) than valid cues (363 ms). Cue type and validity interacted (*F*(1, 967) = 101.218, *p* < 0.001, *η*_p_^2^ = 0.095), such that the difference between valid and invalid cues was larger following arrow (35 ms) than gaze cues (3 ms), *F*(1, 972) = 1141.087, *p* < 0.001, *η*_p_^2^ = 0.540. However, both arrow (*F*(1, 972) = 1961.046, *p* < 0.001, *η*_p_^2^ = 0.669) and gaze (*F*(1, 972) = 21.565, *p* < 0.001, *η*_p_^2^ = 0.022) cues produced significant effects. Cue type interacted with group (*F*(1, 967) = 6.083, *p* = 0.014, *η*_p_^2^ = 0.006), such that the difference between trials following arrow vs. gaze cues was larger for autistic (9 ms) than non‐autistic (7 ms) participants (*t*(971) = 2.209, *p* = 0.027). Cue validity also interacted with group (*F*(1, 967) = 5.873, *p* = 0.016, *η*_p_^2^ = 0.006), such that the validity effect was greater for autistic (20 ms) than non‐autistic (17 ms) participants, *t*(971) = 2.446, *p* = 0.015. Moreover, cue validity interacted with gender (*F*(1, 967) = 6.518, *p* = 0.011, *η*_p_^2^ = 0.007) and in a three‐way interaction with cue type and gender (*F*(1, 967) = 9.325, *p* = 0.002, *η*_p_^2^ = 0.010), such that the difference in the magnitude of the arrow and gaze cueing effects was larger among women (34 ms) than men (29 ms), *t*(971) = 3.087, *p* = 0.002. All other *F* ≤ 4.243; all other *p* ≥ 0.040.

*Time‐series analysis*: With matched subgroups (*N* = 243 per group), we examined the magnitude of the validity effect over the course of trials using a walking average with bins of 46 trials. We conducted a repeated‐measures ANOVA with bin as a within‐subjects factor and group as a between‐subjects factor for each cue type (respectively). Due to violation of the assumption of sphericity, Huynh‐Feldt corrections were applied. Neither group (*F*(1, 485) = 0.743, *p* = 0.389, *η*_p_^2^ = 0.002) nor bin number (*F*(46, 22,310) = 0.391, *p* = 0.752, *η*_p_^2^ = 8.055 × 10^−4^) had a significant main effect on the magnitude of the gaze cueing effect, and they did not interact (*F*(46, 22,310) = 0.485, *p* = 0.686, *η*_p_^2^ = 9.984 × 10^−4^). For the arrow cueing effect, group had a significant main effect (*F*(1, 485) = 5.718, *p* = 0.017, *η*_p_^2^ = 0.012). However, bin number did not (*F*(46, 22,310) = 0.986, *p* = 0.395, *η*_p_^2^ = 0.002), nor did it interact with group (*F*(46, 22,310) = 1.257, *p* = 0.288, *η*_p_^2^ = 0.003).

### 3.5 Overarching Analyses

*ADHD*: We reran all primary analyses (RT for the go/no‐go and arrow/gaze cueing tasks, maximum span for the chessboard task, and total completion time for the TMT) as before but within the autism group and with co‐occurring ADHD (instead of group) as a cofactor. Individuals without data on co‐occurring ADHD were excluded from these analyses. The number of autistic individuals with co‐occurring ADHD was 40/335 on the Go/No‐Go task, 56/478 on the TMT, 45/357 on the chessboard task, and 56/472 on the arrow/gaze cueing task. ADHD diagnostic status had no main effect in any analysis, nor did it interact with any terms (*F* ≤ 1.030; all other *p* ≥ 0.311).

*ICAR*: We conducted sensitivity analyses, removing ICAR as a covariate for each analysis in which it was previously included. Doing so had no effect on the previously reported significance of the main effects or interactions of the group.

## 4 Discussion

Our four‐pronged investigation of EF differences among autistic adults has revealed some notable differences between groups but also many similarities. RTs among autistic participants were generally longer, and we found tentative evidence for disparities in spatial working memory and the effects of cues on attention. However, nonsignificant differences between groups were more common (characterizing our primary measures of inhibition, working memory, and cognitive flexibility, as well as many more specific analyses), and most effects of group that were detected were quite modest. What is more, the demographic factors we included in our analyses (like age, gender, estimated IQ, and ADHD status) did not influence the differences between groups, nor did performance fluctuate over time in markedly different patterns (except for the chessboard task). This lack of major differences between groups is in stark contrast to the effects of age, which were very pronounced in our primary measures of inhibition, cognitive flexibility, and spatial working memory (as well as many secondary analyses), all of which revealed the anticipated effects upon participants overall. This raises questions about the implications of the differences we did detect as well as the reasons that we did not replicate others.

The most consistent difference detected between groups was generally longer RTs across conditions for autistic individuals in each experiment (besides the TMT). This finding is consistent with our previously published findings with the same cohort showing slower RTs among the autistic participants on a range of emotion recognition tasks (Jertberg et al. 2025). This also resonates with two meta‐analyses showing slower RTs among autistic participants across a range of different tasks, including many related to EF (Velikonja et al. 2019; Zapparrata et al. 2023). On the other hand, it contrasts with Ferraro (2016), which found no difference in RT between groups. However, the Brinley plot analysis they employed had no means of accounting for the disparities in age between groups in the studies included. Given the significant effects of age on RT that we detected across tasks, this may explain why their conclusions differed from the other meta‐analyses and our own findings.

The question, then, is at what level these differences in RT emerge. The first possibility is the sensory input level. Both behavioral and neurological visual processing differences have been detected in autism (Farashi et al. 2023; Samson et al. 2011); however, at this level, there seems to be more evidence of *greater* efficiency among autistic individuals, at least for simple stimuli. Other studies show meta‐cognitive differences in autism (Van der Plas et al. 2023), which could translate into a different time course of decision making (i.e., reaching the confidence threshold necessary to give a response). Autistic individuals may also prioritize accuracy over speed to a greater degree than non‐autistic ones or simply exhibit slower cognitive processing speeds (Karalunas et al. 2018). Finally, autistic individuals have shown reduced coordination and general slowing across a range of motor tasks, which could suggest that at least some part of these differences occurs at the mouse/keyboard response level (Fournier et al. 2010; Morrison et al. 2018). With all of this in mind, it would be premature to conclude that these disparities in RT are solely due to differences in EF, and further research should focus on unraveling these various levels of differences to determine their respective contributions to the trends seen in the literature. Drift diffusion models may be of use to translate behavioral data into components of cognitive processing (Karalunas et al. 2018; Ratcliff and Rouder 1998).

Beyond RT, autistic participants showed a greater propensity to errors on the first color of blocks they had to respond to on the chessboard task (suggesting potential spatial working memory differences). They also showed a slightly lower maximum span than non‐autistic participants, in line with the implications of the higher error rate. However, this effect did not survive Bonferroni correction. This may be due to the highly conservative approach we took to correction (an interpretation supported by the fact that, in the time‐series analysis for this task, the interaction between group and trial number did survive Bonferroni correction, with significant differences emerging at specific points in the experiment). Still, the magnitude of the effect of group on maximum span was also quite small, so any difference in spatial working memory it implies is likely to be rather modest. This is further supported by the outcomes of our Bayesian follow‐up analyses (reported in the Supporting Information), in which the model most likely to explain differences in maximum span did not include group, and it was nearly an order of magnitude more likely than the best model that did.

There were also differences between groups in the influence of previous trial type on the rate of directional errors in the Go/No‐Go task (which were more frequent following go trials). The fact that autistic participants were less affected by the previous trial type could be taken as a reflection of better inhibition (i.e., less likelihood to make a mistake by repeating the key pressed on a previous go trial). However, the error rate was generally very low (< 1% of trials). Additionally, the rate of commission errors and the influence of previous trial type on RT are the two most commonly analyzed metrics of inhibition, and no differences between groups were observed in either. For these reasons, the sum of the evidence appears to weigh against major differences in inhibition between groups.

Finally, on the arrow/gaze cueing task, the effects of cue type and validity were both larger for autistic participants. At face value, a greater difference between cue types might be taken as further evidence of the reduced attentional preference for social stimuli seen among autistic individuals in other experiments (Chita‐Tegmark 2016; Hedger et al. 2020). However, differences between valid and invalid cues of both types were numerically larger among autistic participants (see Table S5). Additionally, the hypothesized three‐way interaction of group, cue type, and cue validity (which would have spoken directly to the magnitude of social vs. nonsocial attentional orienting) was not significant. As such, while our results suggest that autistic individuals may be more susceptible to cueing effects in general, the importance of social information to this difference deserves further investigation.

What we did not find in this series of experiments may be more enlightening than what we did. Not only was there a lack of convincing evidence for differences in any of the primary measures of the three domains of EF (confirmed by our follow‐up Bayesian analyses, seen in the Supporting Information, which consistently show that the best models accounting for the effects seen in these variables did not include group), many of the variables that may have been expected to moderate the potential differences between groups were not found to do so in our analyses. While age and gender had significant effects of their own, they did not interact with group (with the sole exception of the group × gender interaction on the chessboard task). This aligns with the similar patterns of findings in our age‐ and gender‐matched time‐series analyses. Additionally, given the intimate relationship between IQ and EF (Davis et al. 2011; Duggan and Garcia‐Barrera 2015), there is justifiable concern that controlling for one variable might also control for the other. However, our sensitivity analyses revealed no differences in any of the outcomes of comparisons between groups with or without estimated IQ as a covariate. We also detected no differences in performance between autistic participants who did and did not report co‐occurring ADHD diagnoses. However, conclusions should be tempered here, given that we relied upon self‐report of ADHD diagnoses, and ADHD is often missed in autistic individuals (and vice versa). Due to the risk that some autistic participants may have had undiagnosed ADHD, it would be premature to conclude from this study alone that autistic individuals with/without co‐occurring ADHD show comparable EF abilities. Future research should investigate the topic more thoroughly by verifying ADHD diagnostic status and confirming a significant difference in symptoms of ADHD between groups.

If not these variables, then what explains why we do not see differences in the domains of EF where prior meta‐analyses have detected evidence for impairment among autistic individuals? Firstly, it is worth noting that even in these meta‐analyses, considerable heterogeneity in outcomes was detected. Nonsignificant differences between groups have been reported previously in all three domains, even with the same paradigms in the case of the trail making and Go/No‐Go tasks (Geurts et al. 2009; Happé et al. 2006; Hlavatá et al. 2018; Morrison et al. 2018; Ozonoff and Strayer 2001; Torenvliet et al. 2023). It is also notable that our sample is considerably larger than that of the vast majority of other studies. Moreover, Demetriou et al. (2017) noted that studies involving self‐ or carer‐reported ratings had higher effect sizes than experimental tasks across EF domains. Similarly, Leung and Zakzanis (2014) found that only the shift subscale of the self‐report version of the Behavioral Rating Inventory of EF could reliably distinguish between autistic and non‐autistic individuals in terms of cognitive flexibility. This was in contrast to the range of performance‐based studies they included, several of which employed the TMT. This was also seen within samples in Davids et al. (2016) and Geurts et al. (2020), where older autistic adults reported EF difficulties that were not supported by measurable objective differences in cognitive tasks.

There are two potential explanations for these discrepancies, which are not mutually exclusive: (1) subjective reports might underestimate the EF abilities of autistic individuals (and/or overestimate the abilities of those without autism), and (2) experimental tasks might lack the ecological validity necessary to replicate the conditions that lead to EF difficulties among autistic individuals. While it is difficult to speak to the first point given the possibility of the second, it is worth noting that research with other clinical and nonclinical groups has detected similar discrepancies between subjective and objective measures (Groenman et al. 2022; Mazza et al. 2021). In one study, it was found that while depressed individuals appeared to underestimate their own cognitive abilities, a healthy comparison group overestimated theirs (Schwert et al. 2018). This is particularly notable given the higher rates of depression among autistic individuals, coupled with their higher rates of alexithymia, both of which may cloud introspection on cognitive abilities (Kinnaird et al. 2019; Poquérusse et al. 2018). Similarly, in a study with autistic adults, it was found that subjective cognitive complaints showed no relationship with objective cognitive performance (besides a weak association for visual memory performance), whereas they were strongly associated with depression (Torenvliet et al. 2024). These findings might raise questions as to the reliability of the subjective measures that detect the largest differences between groups in EF function ability. Conversely, tasks isolating specific components of EF may fail to reproduce the features that lead to the real‐life difficulties in EF being reported by autistic individuals, such as ambiguity, lack of structure, and background distractions (Kenworthy et al. 2008). Comparisons between two imperfect measures are not suited to resolve this debate, and efforts to enhance the ecological validity of EF measures often reduce the specificity with which they target the construct in question. Still, pursuing greater ecological validity is a clear first step toward bridging the gap between subjective and objective findings.

In any case, discrepancies between subjective and objective measures of EF cannot fully explain the differences between our findings and previous research. Demetriou et al. (2017) conducted a sensitivity analysis without questionnaire data, and, although effect sizes dropped considerably, they still found differences between groups in performance across a wide range of EF tasks. This was also the case in Lai et al. (2017), which meta‐analyzed data exclusively from neuropsychological measures. However, it is worth noting that Demetriou et al. (2017) showed smaller differences between autistic and non‐autistic individuals in their adult subgroup analysis and Lai et al. (2017) only studied children and adolescents. In line with this trend, Wang et al. (2024) reported a comparatively small difference between autistic and non‐autistic adults over 40 across objective EF measures. Notably, the difference was largest in working memory tasks, where we also detected differences in error rates and span at certain time‐points in our experiment. Findings were highly heterogeneous and nonsignificant overall for cognitive flexibility, in contrast (although there was a significant effect for the TMT). So, while findings with adults are less consistent, there is some evidence of differences in EF performance, including in similar tasks/domains to those in our experiments.

Changes over time in the makeup of the autistic population (and resulting differences in sampling) may also be relevant to these divergent outcomes. Demetriou et al. (2017) found a significant negative influence of the year of publication on differences between groups, in line with the findings of Rødgaard et al. (2019) that effect sizes across cognitive domains (including many measures of EF) have declined over the past two decades. Both authors attribute this trend to a diversification of the autism spectrum over time, which may be related to broadening diagnostic criteria and an increase in adult diagnoses. Given that our sample is disproportionately comprised of adults diagnosed later in life (with high IQs relative to the norms for autistic individuals), one might suspect that it may be biased toward individuals with less pronounced autistic traits, as they have a negative association with the age of diagnosis (Hrdlicka et al. 2023). However, the mean age of diagnosis is not commonly reported, so we cannot compare our sample to others on this basis. Additionally, the average AQ‐28 score of our autistic sample (above 82 on all measures) was significantly different from that of our non‐autistic sample and well above the screening cut‐off of 65 suggested by the creators of the questionnaire (Hoekstra et al. 2011). We chose not to apply this cut‐off because we wanted to recruit community samples that were as representative as possible of the overlapping distributions of autistic traits in the autistic and non‐autistic populations, but the disparity between groups in AQ scores was vast nonetheless. So, although our sample is not perfectly representative of the full autism spectrum, there is no evidence to suggest that autistic traits are not sufficiently pronounced to detect associated differences.

Due to these particularities of our sample, conclusions from our data should be circumscribed to autistic adults without intellectual disability. Additionally, the general lack of performance differences cannot be taken as definitive evidence that these individuals do not face challenges with EF in daily life. Moreover, we chose a fixed order of tasks to balance the larger battery comprising them and to ensure that any possible differences in the degree of fatigue a participant experienced on a given task were not due to task order, allowing us to isolate differences relevant to the group. We also divided the battery into two brief sessions to minimize any such fatigue effects. Still, the fixed order does introduce a potential confound: that differences in fatigue (or other forms of contamination across tasks), rather than ability, might drive performance differences between groups, particularly in the later tasks. However, the similar performance over time between groups suggests that fatigue and carryover effects were experienced at a similar level. Additionally, the groups were not found to differ in most of our primary measures, so it is unlikely that this potential confound played a major role in the pattern of results that emerged.

## 5 Conclusions

The wide range of tasks and analytical approaches we employed provides a diverse selection of evidence that autistic adults can reach similar levels of performance to non‐autistic ones in tasks designed to isolate specific EF abilities. This may imply that by making environments in work and school more suitable for autistic individuals and providing structure, explicit instructions, and accommodations for slower processing/response speed, some of the difficulties they report might be ameliorated. Moreover, it challenges theories that depend upon universal differences in basic components of EF. Finally, the differences that we did detect in RTs, cueing effects, and propensity to working memory errors indicate potential problem areas that deserve further research with more naturalistic paradigms.

## Conflicts of Interest

The authors declare no conflicts of interest.

## Supporting information


Data S1.

## Data Availability

The data that support the findings of this study are available on request from the corresponding author. The data are not publicly available due to privacy or ethical restrictions.

## Endnotes

^1^
We alternate between person‐first and identity‐first language to address the varying preferences of the international autism community, as recommended in Buijsman et al. (2023).

^2^
Note that for this experiment (and those that follow), we deviated from our pre‐registered plan to conduct a time‐series analysis on all dependent variables for which significant group differences were detected, choosing instead to focus on the primary effect of interest. This is because many variables, such as certain error types, did not occur frequently enough to properly analyze in this fashion. Additionally, such an approach would lack sensitivity to differences that might emerge at certain points in the experiment that were lost in the overall means. Finally, it would fail to capture the underlying construct in question in some cases. For example, in this experiment, it would mean investigating raw RT data over the course of the experiment, rather than the magnitude of the inhibition effect the task was designed to capture.
