# Supplementary material for: Slow but Steady: Similarities and Differences in Executive Functioning Between Autistic and Non‐Autistic Adults
Source: Autism Res. 2025 Mar 13;18(4):802–19. doi: 10.1002/aur.70015 (PMC12015807; doi:10.1002/aur.70015)
Supplement: Supplementary file 1 — Data S1. [file AUR-18-802-s001.docx]

**Supplementary Materials**

**Slow but Steady: Similarities and Differences in Executive Functioning Between Autistic and Non-autistic Adults**

Robert Jertberg^1*^, Sander Begeer^1^, Hilde M. Geurts^2,3^, Bhismadev Chakrabarti^4,5,6^, and Erik Van der Burg^1^

^1^ Section Clinical Developmental Psychology, Vrije Universiteit

Amsterdam and the Netherlands and Amsterdam Public Health

Research Institute, The Netherlands

^2^Dutch Autism and ADHD research Center (d’Arc), Brain & Cognition, Department of Psychology, Universiteit van Amsterdam, The Netherlands

^3^Leo Kannerhuis (Youz/Parnassiagroup), The Netherlands

^4^Centre for Autism, School of Psychology and Clinical Language Sciences, University of Reading, UK

^5^India Autism Center, Kolkata, India

^6^Department of Psychology, Ashoka University, India

*Corresponding author: Robert Jertberg

jertbergiii@gmail.com

**
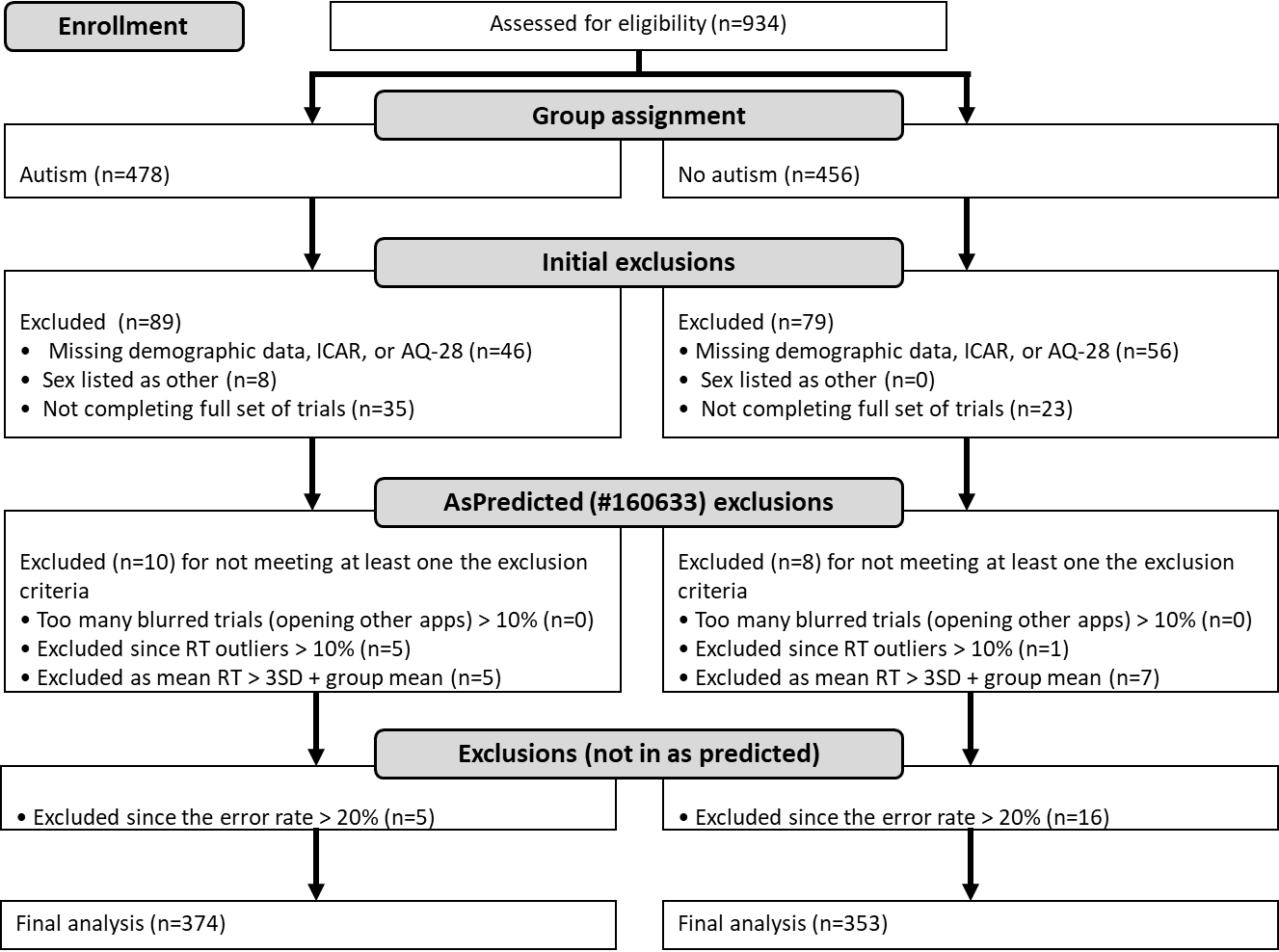
**

Supplementary Figure 1: flowchart depicting reasons for participant exclusion in the Go/No-Go Task.


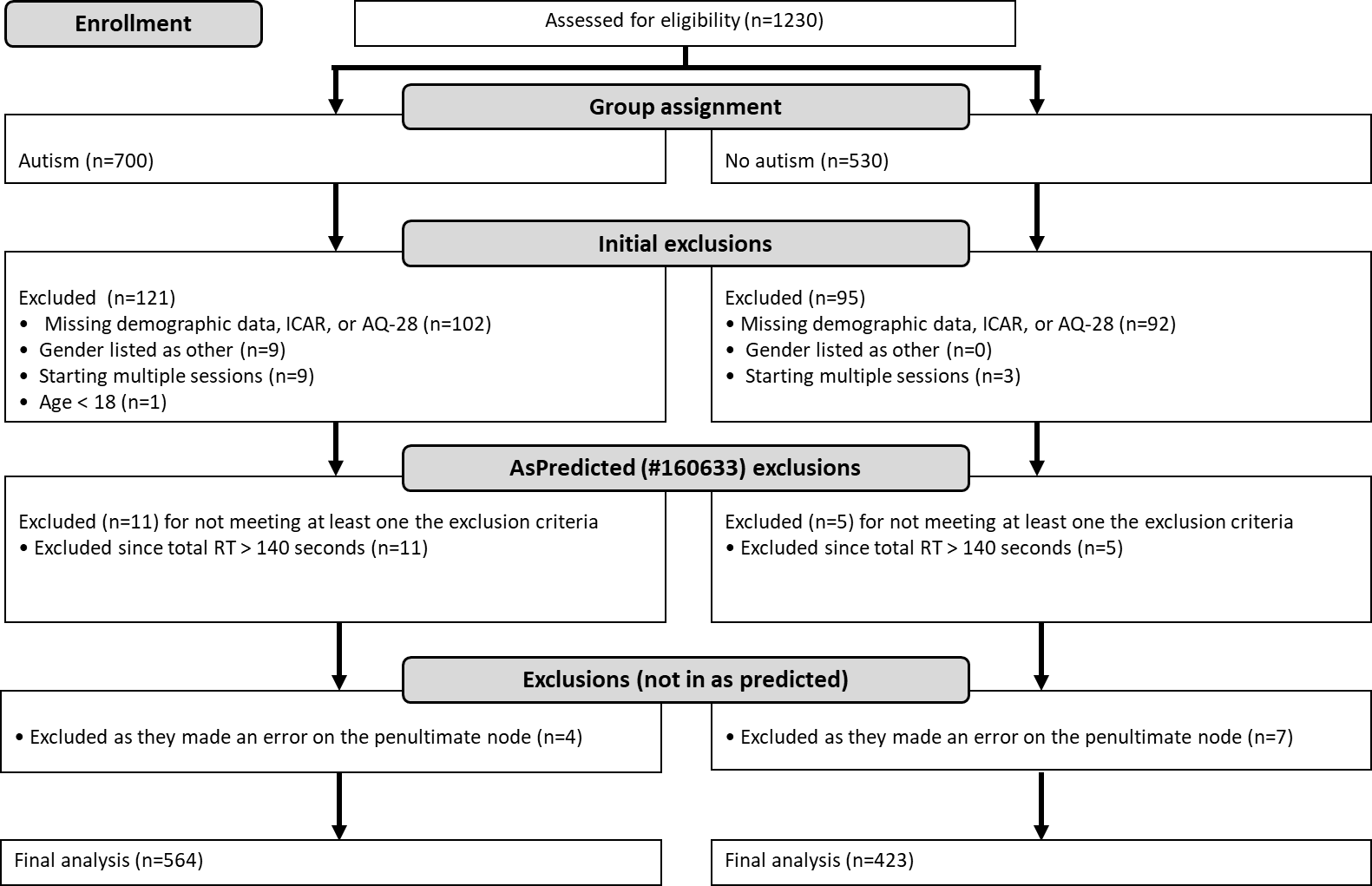


Supplementary Figure 2: flowchart depicting reasons for participant exclusion in the Trail Making Task.


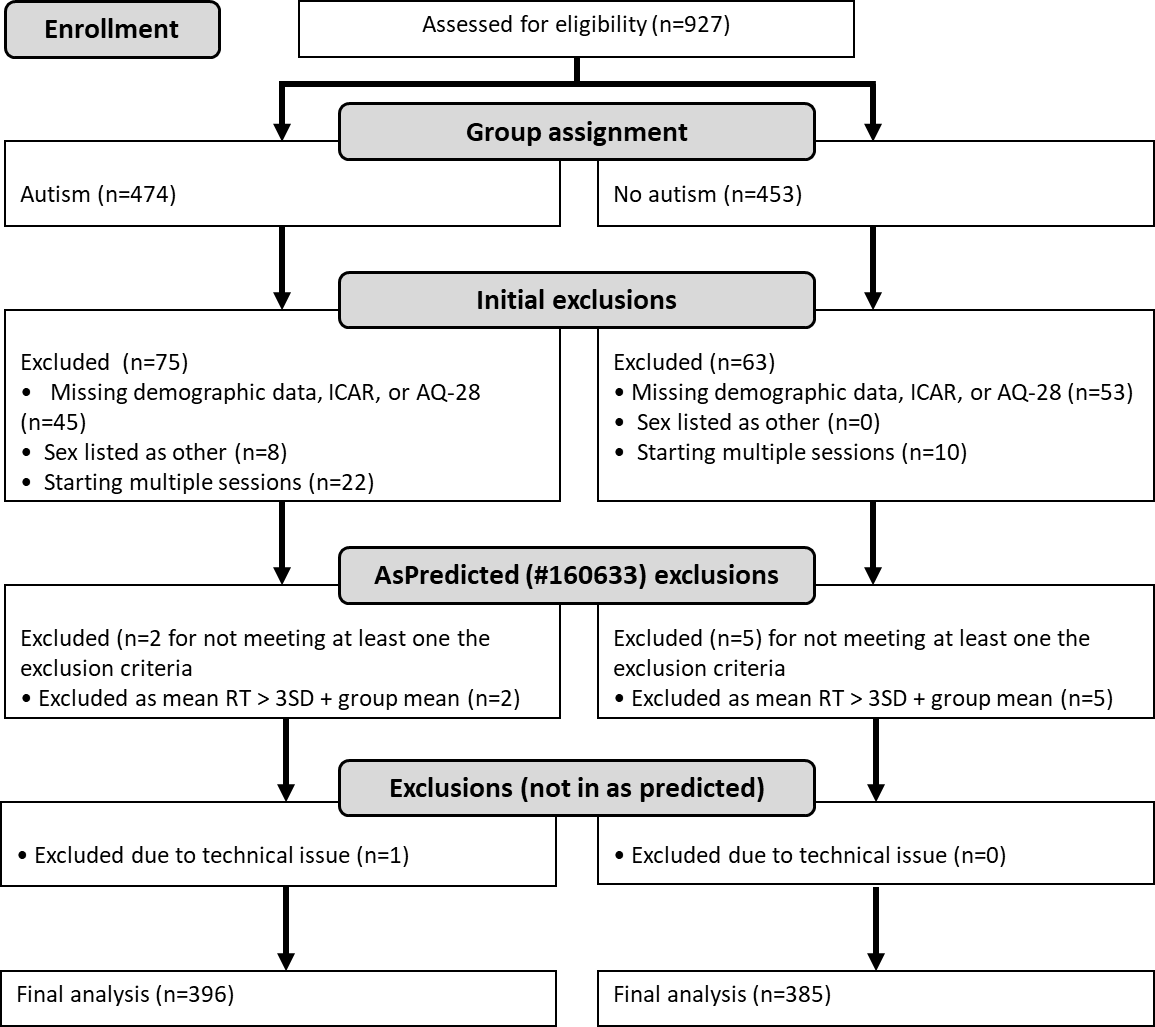


Supplementary Figure 3: flowchart depicting reasons for participant exclusion in the Chessboard Task.

**
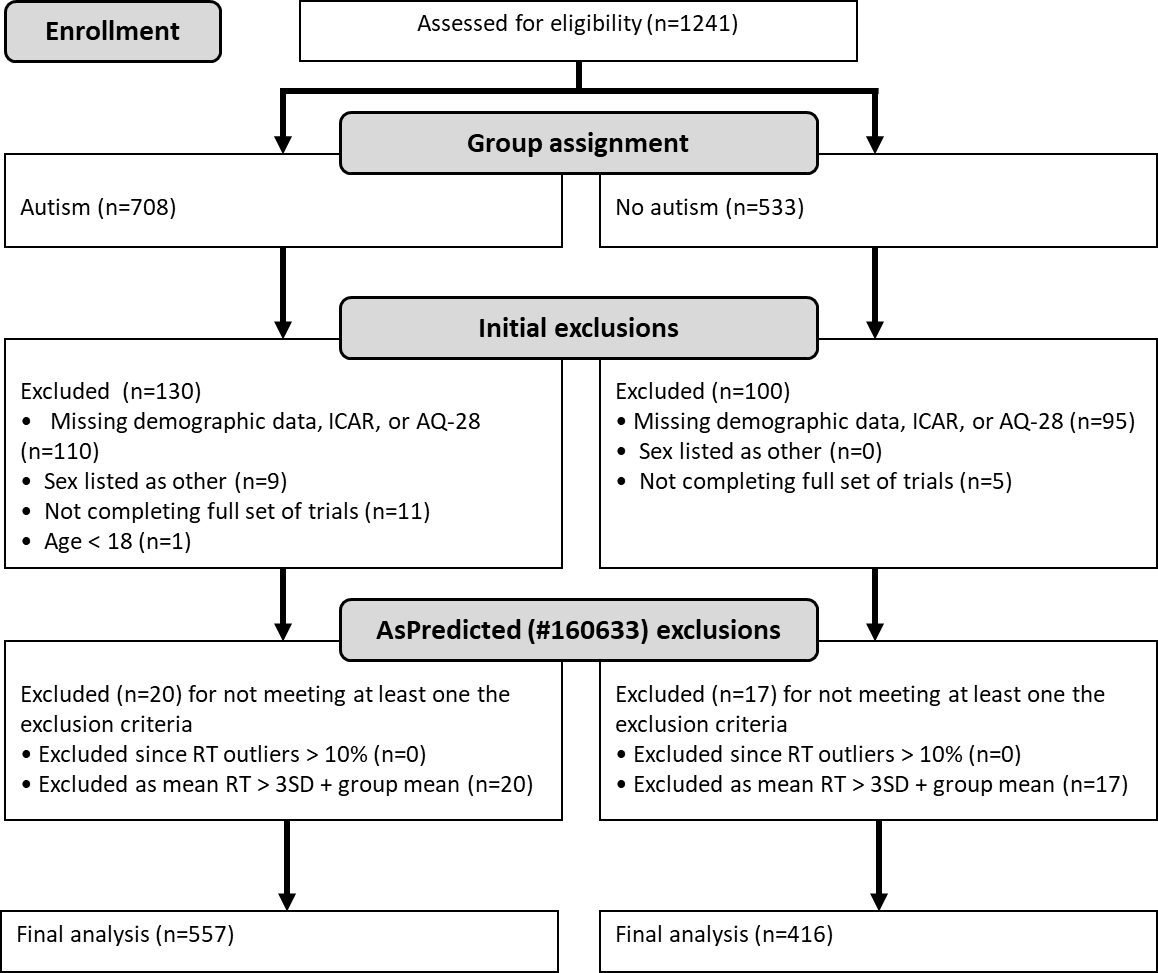
**

Supplementary Figure 4: flowchart depicting reasons for participant exclusion in the Arrow/Gaze Cueing Task.

**S1 Continuation of Results**

RT, primary measures of each construct, and time series analyses are reported in the results section of the main manuscript. Below, one can find the results of the analyses of all other (preregistered) dependent measures, which are also depicted in the figures from the main manuscript.

**Experiment 1: Inhibition (Go/No-Go Task)**

Note: As we analyzed three dependent variables, alpha was set to .017.

*Mean proportion of directional errors.* The effect of group was not significant (*F*(1, 721) = 0.315, *p* = .575, η_p_^2^ = 4.366 x 10^-4^). Age was negatively related to the rate of directional errors (*F*(1, 721) = 13.054, *p* = .008, η_p_^2^ = .018). Previous trial type had a significant effect (*F*(1, 721) = 17.704, *p* < .001, η_p_^2^ = .024), such that directional errors were more common following go trials (1.0 %) than no-go trials (0.6 %). Previous trial type also interacted with age (*F*(1, 721) = 8.317, *p* = .004, η_p_^2^ = .011), such that the difference in error rates between previous trial types was greater among younger participants (*r* = -0.178, *p* < .001). Moreover, previous trial type interacted with group (*F*(1, 721) = 7.407, *p* = .007, η_p_^2^ = .010), such that the autistic individuals exhibited a smaller difference between previous trial types (0.2%) than non-autistic ones (0.7%), *t*(725) = -4.579, *p* < .001. All other *F*  ≤ 5.394; all other *p* ≥ .020.

*Mean proportion of commission errors*. Group did not have a significant main effect (*F*(1, 721) = 1.967, *p* = .161, η_p_^2^ = .003), nor did it interact with previous trial type (*F*(1, 721) = 0.757, *p* = .384, η_p_^2^ = .001). Age had a negative relationship with the rate of commission errors (*F*(1, 721) = 18.107, *p* < .001, η_p_^2^ = .024). Previous trial type had a significant effect (*F*(1, 721) = 40.650, *p* < .001, η_p_^2^ = .053), with participants being more likely to make commission errors following Go trials (2.9%) than No-Go trials (1.1%). Age and previous trial type also interacted (*F*(1, 721) = 15.659, *p* < .001, η_p_^2^ = .021), with age correlating negatively with the difference in error rates between trials following Go trials vs No-Go trials (*r* = -0.185, *p* < .001). All other *F*  ≤ 1.967; all other *p* ≥ .161.

*Mean proportion of omission errors.* Due to the fact that omission errors only occurred on 0.02% of trials, we excluded them from further analyses.

*Motor priming analysis.* To evaluate the influence of inter-trial motor effects, we conducted a repeated measures ANOVA on the mean RT of correct Go trials following other correct Go trials with the direction of the previous trial relative to the current one (repetition or switch) as a repeated measures factor, group and gender as between subjects factors, and age and ICAR score as covariates. The previously reported main effect of group remained significant in this analysis (*F*(1, 721) = 9.610, *p* = .002, η_p_^2^ = .013), but group did not interact with previous trial type (*F*(1, 721) = 4.319, *p* = .038, η_p_^2^ = .006). Previous trial type had a significant main effect (*F*(1, 721) = 13.816, *p* < .001, η_p_^2^ = .019), with participants responding more slowly when the direction switched between trials (408 ms) than when it remained the same (396 ms). The main effects of age (*F*(1, 721) = 297.451, *p* < .001, η_p_^2^ = .292) and ICAR (*F*(1, 721) = 14.551, *p* < .001, η_p_^2^ = .020) also remained significant in this analysis. All other *F*  ≤ 4.295; all other *p* ≥ .039.

*Bayesian follow-up.* As we did not find a significant group difference in our primary measure of inhibition (the interaction between previous trial type and group on RT), we conducted a Bayesian follow-up ANOVA with the magnitude of the inhibition effect (i.e., the difference in RT between trials following No-Go vs Go trials) as the dependent variable, group and gender as cofactors, and age and ICAR as covariates. For this and subsequent Bayesian analyses, we adopted uniform model priors and default priors on coefficients (*r* scale prior width = 0.5 for fixed effects and 0.354 for covariates). Compared to the null hypothesis, the best model was offered by age alone (BF_10_ = 1.096 x 10^12^). The model including group as well as age was less likely to explain the data, with a BF_10_ of 6.988 x 10^11^. See Supplementary Table 1 for the full results of the Bayesian analysis.

Additionally, as the rate of commission errors is also a frequently reported primary measure of inhibition, we also conducted a Bayesian follow-up ANOVA with the overall rate of commission errors as the dependent variable, group and gender as cofactors, and age and ICAR as covariates. Compared to the null hypothesis, the best model was offered by age alone (BF_10_ = 3.150 x 10^7^). The model including group as well as age was less likely to explain the data, with a BF_10_ of 1.226 x 10^7^. See Supplementary Table 2 for the full results of the Bayesian analysis.

Supplementary Table 1: Results of a Bayesian analysis of covariance on the difference in reaction time between trials following No-Go vs Go trials in the Go/No-Go task with group and gender as between subject variables and age and International Cognitive Ability Resource intelligence quotient scores as covariates. This analysis was conducted using uniform model priors and default priors on coefficients (*r* scale prior width = 0.5 for fixed effects and 0.354 for covariates).

| **Model Comparison** | | | | | | | | | | | |
| --- | --- | --- | --- | --- | --- | --- | --- | --- | --- | --- | --- |
| **Models** | | **P(M)** | | **P(M\|data)** | | **BF_M_** | | **BF_10_** | | **error %** | |
| Null model |  | 0.050 |  | 3.454×10^-13^ |  | 6.563×10^-12^ |  | 1.000 |  |  |  |
| Age |  | 0.050 |  | 0.378 |  | 11.567 |  | 1.096×10^+12^ |  | 1.392×10^-4^ |  |
| Age + ('group', '') |  | 0.050 |  | 0.241 |  | 6.046 |  | 6.988×10^+11^ |  | 1.109 |  |
| Age + Gender |  | 0.050 |  | 0.152 |  | 3.407 |  | 4.402×10^+11^ |  | 2.052 |  |
| Age + ICAR_measure_prop |  | 0.050 |  | 0.059 |  | 1.197 |  | 1.716×10^+11^ |  | 0.004 |  |
| Age + Gender + ('group', '') |  | 0.050 |  | 0.054 |  | 1.083 |  | 1.562×10^+11^ |  | 1.349 |  |
| Age + ICAR_measure_prop + ('group', '') |  | 0.050 |  | 0.045 |  | 0.889 |  | 1.294×10^+11^ |  | 1.262 |  |
| Age + Gender + ('group', '') + Gender ✻  ('group', '') |  | 0.050 |  | 0.029 |  | 0.576 |  | 8.513×10^+10^ |  | 1.792 |  |
| Age + Gender + ICAR_measure_prop |  | 0.050 |  | 0.026 |  | 0.498 |  | 7.392×10^+10^ |  | 1.467 |  |
| Age + Gender + ICAR_measure_prop + ('group', '') |  | 0.050 |  | 0.010 |  | 0.191 |  | 2.884×10^+10^ |  | 1.731 |  |
| Age + Gender + ICAR_measure_prop + ('group', '') + Gender ✻  ('group', '') |  | 0.050 |  | 0.005 |  | 0.102 |  | 1.546×10^+10^ |  | 2.411 |  |
| Gender + ('group', '') + Gender ✻  ('group', '') |  | 0.050 |  | 1.562×10^-8^ |  | 2.968×10^-7^ |  | 45217.823 |  | 3.858 |  |
| ('group', '') |  | 0.050 |  | 1.161×10^-8^ |  | 2.206×10^-7^ |  | 33616.127 |  | 8.012×10^-7^ |  |
| Gender + ICAR_measure_prop + ('group', '') + Gender ✻  ('group', '') |  | 0.050 |  | 2.400×10^-9^ |  | 4.560×10^-8^ |  | 6948.330 |  | 3.492 |  |
| ICAR_measure_prop + ('group', '') |  | 0.050 |  | 1.630×10^-9^ |  | 3.096×10^-8^ |  | 4718.143 |  | 1.986 |  |
| Gender + ('group', '') |  | 0.050 |  | 1.167×10^-9^ |  | 2.218×10^-8^ |  | 3379.575 |  | 1.259 |  |
| Gender + ICAR_measure_prop + ('group', '') |  | 0.050 |  | 1.777×10^-10^ |  | 3.376×10^-9^ |  | 514.407 |  | 1.820 |  |
| Gender |  | 0.050 |  | 1.041×10^-13^ |  | 1.979×10^-12^ |  | 0.301 |  | 0.065 |  |
| ICAR_measure_prop |  | 0.050 |  | 3.975×10^-14^ |  | 7.553×10^-13^ |  | 0.115 |  | 0.002 |  |
| Gender + ICAR_measure_prop |  | 0.050 |  | 1.299×10^-14^ |  | 2.467×10^-13^ |  | 0.038 |  | 1.135 |  |
|  | | | | | | | | | | | |

Supplementary Table 2: Results of a Bayesian analysis of covariance on the rate of errors of commission in the Go/No-Go task with group and gender as between subject variables and age and International Cognitive Ability Resource intelligence quotient scores as covariates. This analysis was conducted using uniform model priors and default priors on coefficients (*r* scale prior width = 0.5 for fixed effects and 0.354 for covariates).

| **Model Comparison** | | | | | | | | | | | |
| --- | --- | --- | --- | --- | --- | --- | --- | --- | --- | --- | --- |
| **Models** | | **P(M)** | | **P(M\|data)** | | **BF_M_** | | **BF_10_** | | **error %** | |
| Null model |  | 0.050 |  | 1.800×10^-8^ |  | 3.419×10^-7^ |  | 1.000 |  |  |  |
| Age |  | 0.050 |  | 0.567 |  | 24.873 |  | 3.150×10^+7^ |  | 0.003 |  |
| ('group', '') + Age |  | 0.050 |  | 0.221 |  | 5.381 |  | 1.226×10^+7^ |  | 0.962 |  |
| Age + ICAR_measure_prop |  | 0.050 |  | 0.084 |  | 1.752 |  | 4.691×10^+6^ |  | 9.386×10^-4^ |  |
| Gender + Age |  | 0.050 |  | 0.051 |  | 1.028 |  | 2.851×10^+6^ |  | 2.163 |  |
| ('group', '') + Age + ICAR_measure_prop |  | 0.050 |  | 0.035 |  | 0.694 |  | 1.957×10^+6^ |  | 1.514 |  |
| ('group', '') + Gender + Age |  | 0.050 |  | 0.024 |  | 0.472 |  | 1.347×10^+6^ |  | 1.581 |  |
| Gender + Age + ICAR_measure_prop |  | 0.050 |  | 0.008 |  | 0.147 |  | 427798.772 |  | 4.299 |  |
| ('group', '') + Gender + Age + ('group', '') ✻  Gender |  | 0.050 |  | 0.005 |  | 0.091 |  | 265755.220 |  | 2.463 |  |
| ('group', '') + Gender + Age + ICAR_measure_prop |  | 0.050 |  | 0.004 |  | 0.074 |  | 216155.975 |  | 1.640 |  |
| ('group', '') + Gender + Age + ICAR_measure_prop + ('group', '') ✻  Gender |  | 0.050 |  | 7.732×10^-4^ |  | 0.015 |  | 42965.934 |  | 2.417 |  |
| ('group', '') |  | 0.050 |  | 1.073×10^-5^ |  | 2.040×10^-4^ |  | 596.478 |  | 4.146×10^-5^ |  |
| ('group', '') + Gender |  | 0.050 |  | 2.072×10^-6^ |  | 3.936×10^-5^ |  | 115.111 |  | 1.027 |  |
| ('group', '') + ICAR_measure_prop |  | 0.050 |  | 1.334×10^-6^ |  | 2.534×10^-5^ |  | 74.102 |  | 2.008 |  |
| ('group', '') + Gender + ('group', '') ✻  Gender |  | 0.050 |  | 2.485×10^-7^ |  | 4.721×10^-6^ |  | 13.807 |  | 5.494 |  |
| ('group', '') + Gender + ICAR_measure_prop |  | 0.050 |  | 2.274×10^-7^ |  | 4.320×10^-6^ |  | 12.635 |  | 1.651 |  |
| ('group', '') + Gender + ICAR_measure_prop + ('group', '') ✻  Gender |  | 0.050 |  | 3.119×10^-8^ |  | 5.925×10^-7^ |  | 1.733 |  | 4.825 |  |
| ICAR_measure_prop |  | 0.050 |  | 1.906×10^-9^ |  | 3.622×10^-8^ |  | 0.106 |  | 0.002 |  |
| Gender |  | 0.050 |  | 1.682×10^-9^ |  | 3.195×10^-8^ |  | 0.093 |  | 0.202 |  |
| Gender + ICAR_measure_prop |  | 0.050 |  | 1.753×10^-10^ |  | 3.332×10^-9^ |  | 0.010 |  | 2.031 |  |
|  | | | | | | | | | | | |

**Experiment 2: Cognitive Flexibility (Trail Making Task)**

Note: As there were two dependent variables, alpha was set to .025.

*Mean response time per node*. We pre-registered that we would repeat the previous analysis, but with the mean time required to click a node (after removing outlier trials in which participants took longer than 30 seconds, following the threshold determined by Agelink van Rentergem et al., 2020). As these outliers were extremely rare (occurring on 0.05% of nodes), we decided to exclude this analysis.

*Errors^[[1]](#footnote-1)^.* Group did not have a significant main effect (*F*(1, 981) = 0.801, *p* = .371, η_p_^2^ = 8.157 x 10^-4^), nor did it interact with part (*F*(1, 981) = 0.113, *p* = .737, η_p_^2^ = 1.154 x 10^-4^). ICAR score had a negative relationship with combined error rate (*F*(1, 981) = 16.139, *p* < .001, η_p_^2^ = .016). Part had a significant main effect (*F*(1, 981) = 15.770, *p* < .001, η_p_^2^ = .016), with participants making more errors on part B (1.0) than A (0.6). Part also interacted with ICAR score (*F*(1, 981) = 5.728, *p* = .017, η_p_^2^ = .006), with ICAR score correlating negatively (*r* = 0.077, *p* = .016) with the difference in error rates between parts. All other *F* ≤ 4.828; all other *p* ≥ .028.

*Bayesian follow-up.* Because we did not detect a significant group difference in our primary measure of cognitive flexibility (the interaction between part and group on total completion time), we conducted a Bayesian follow-up ANOVA with the magnitude of the difference between parts in total completion time as the dependent variable, group and gender as cofactors, and age and ICAR as covariates. Compared to the null hypothesis, the best model was offered by gender, age, and ICAR score (BF_10_ = 6.157 x 10^18^), and the BF_10_ of the model including those factors as well as group was 1.451 x 10^18^. See Supplementary Table 3 for the full results of the Bayesian analysis.

Supplementary Table 3: Results of a Bayesian analysis of covariance on the difference in total completion time between trail making task B and trail making task B with group and gender as between subject variables and age and International Cognitive Ability Resource intelligence quotient scores as covariates. This analysis was conducted using uniform model priors and default priors on coefficients (*r* scale prior width = 0.5 for fixed effects and 0.354 for covariates).

| **Model Comparison** | | | | | | | | | | | |
| --- | --- | --- | --- | --- | --- | --- | --- | --- | --- | --- | --- |
| **Models** | | **P(M)** | | **P(M\|data)** | | **BF_M_** | | **BF_10_** | | **error %** | |
| Null model |  | 0.050 |  | 1.260×10^-19^ |  | 2.395×10^-18^ |  | 1.000 |  |  |  |
| Gender + Age + ICAR_measure_prop |  | 0.050 |  | 0.776 |  | 65.783 |  | 6.157×10^+18^ |  | 1.135 |  |
| Gender + group + Age + ICAR_measure_prop |  | 0.050 |  | 0.183 |  | 4.253 |  | 1.451×10^+18^ |  | 1.564 |  |
| Gender + group + Age + ICAR_measure_prop + Gender ✻  group |  | 0.050 |  | 0.022 |  | 0.435 |  | 1.776×10^+17^ |  | 2.042 |  |
| Gender + group + ICAR_measure_prop |  | 0.050 |  | 0.013 |  | 0.258 |  | 1.064×10^+17^ |  | 1.198 |  |
| Gender + ICAR_measure_prop |  | 0.050 |  | 0.003 |  | 0.053 |  | 2.205×10^+16^ |  | 0.828 |  |
| Gender + group + ICAR_measure_prop + Gender ✻  group |  | 0.050 |  | 0.003 |  | 0.049 |  | 2.053×10^+16^ |  | 1.652 |  |
| Age + ICAR_measure_prop |  | 0.050 |  | 3.235×10^-5^ |  | 6.146×10^-4^ |  | 2.567×10^+14^ |  | 0.003 |  |
| group + Age + ICAR_measure_prop |  | 0.050 |  | 3.120×10^-6^ |  | 5.929×10^-5^ |  | 2.476×10^+13^ |  | 1.277 |  |
| ICAR_measure_prop |  | 0.050 |  | 7.482×10^-8^ |  | 1.422×10^-6^ |  | 5.937×10^+11^ |  | 0.002 |  |
| group + ICAR_measure_prop |  | 0.050 |  | 5.686×10^-8^ |  | 1.080×10^-6^ |  | 4.512×10^+11^ |  | 0.936 |  |
| Gender + Age |  | 0.050 |  | 2.341×10^-12^ |  | 4.448×10^-11^ |  | 1.858×10^+7^ |  | 1.433 |  |
| Gender + group + Age |  | 0.050 |  | 7.485×10^-13^ |  | 1.422×10^-11^ |  | 5.939×10^+6^ |  | 49.304 |  |
| Gender + group + Age + Gender ✻  group |  | 0.050 |  | 4.737×10^-14^ |  | 9.000×10^-13^ |  | 375846.453 |  | 3.642 |  |
| Gender + group |  | 0.050 |  | 2.505×10^-14^ |  | 4.759×10^-13^ |  | 198751.176 |  | 1.378 |  |
| Gender |  | 0.050 |  | 1.126×10^-14^ |  | 2.139×10^-13^ |  | 89308.765 |  | 2.895×10^-7^ |  |
| Gender + group + Gender ✻  group |  | 0.050 |  | 5.517×10^-15^ |  | 1.048×10^-13^ |  | 43779.298 |  | 8.131 |  |
| Age |  | 0.050 |  | 4.428×10^-17^ |  | 8.414×10^-16^ |  | 351.374 |  | 0.006 |  |
| group + Age |  | 0.050 |  | 3.487×10^-18^ |  | 6.626×10^-17^ |  | 27.670 |  | 1.791 |  |
| group |  | 0.050 |  | 5.297×10^-20^ |  | 1.006×10^-18^ |  | 0.420 |  | 0.050 |  |
|  | | | | | | | | | | | |

**Experiment 3: Working Memory (Chessboard Task)**

Note: As there were five dependent variables, alpha was set to .010.

*Proportion of orange errors.* Note that error rates reflect the mean number of errors per trial divided by the current span, essentially the mean number of errors per button press, assuming they do not click outside of the grid completely. There was a significant main effect of group (*F*(1, 769) = 9.766, *p* = .002, η_p_^2^ = .013), such that autistic participants made more orange errors (0.059) than non-autistic ones (0.051). Gender also had a significant main effect (*F*(1, 769) = 12.777, *p* < .001, η_p_^2^ = .016), such that men (0.061) made more orange errors than women (0.051). ICAR score correlated negatively with the rate at which participants made orange errors (*F*(1, 769) = 7.409, *p* = .007, η_p_^2^ = .010). All other *F* ≤ 1.129; all other *p* ≥ .288.

*Proportion of blue errors.* There was no significant effect of group (*F*(1, 769) = 1.652, *p* = .199, η_p_^2^ = 0.002). Age correlated positively with the rate of blue errors (*F*(1, 769) = 16.871, *p* < .001, η_p_^2^ = .021), whereas ICAR score correlated negatively (*F*(1, 769) = 20.977 *p* < .001, η_p_^2^ = .027). All other *F* ≤ 1.652; all other *p* ≥ .199.

*Proportion of sequence errors.* There was no significant effect of group (*F*(1, 769) = 3.484, *p* = .062, η_p_^2^ = .005). Gender had a significant main effect (*F*(1, 769) = 11.236, *p* = .001, η_p_^2^ = .014), such that men (0.018) made more sequence errors than women (0.012). All other *F* ≤ 3.784; all other *p* ≥ .052.

*Bayesian follow-up.* Because we did not detect a significant difference between groups in our primary measure of spatial working memory (the maximum span), we conducted a Bayesian ANCOVA with it as a dependent variable, group and gender as cofactors, and age and ICAR score as covariates. Compared to the null hypothesis, the best model was offered by age and ICAR score (BF_10_ = 2.652 x 10^31^), and the BF_10_ of the model including those factors as well as group was 9.354 x 10^30^. See Supplementary Table 4 for the full results of the Bayesian analysis.

Supplementary Table 4: Results of a Bayesian analysis of covariance on the maximum working memory span with group and gender as between subject variables and age and International Cognitive Ability Resource intelligence quotient scores as covariates. This analysis was conducted using uniform model priors and default priors on coefficients (*r* scale prior width = 0.5 for fixed effects and 0.354 for covariates).

| **Model Comparison** | | | | | | | | | | | |
| --- | --- | --- | --- | --- | --- | --- | --- | --- | --- | --- | --- |
| **Models** | | **P(M)** | | **P(M\|data)** | | **BF_M_** | | **BF_10_** | | **error %** | |
| Null model |  | 0.050 |  | 1.480×10^-32^ |  | 2.812×10^-31^ |  | 1.000 |  |  |  |
| Age + ICAR_measure_prop |  | 0.050 |  | 0.392 |  | 12.271 |  | 2.652×10^+31^ |  | 8.430×10^-4^ |  |
| Gender + Age + ICAR_measure_prop |  | 0.050 |  | 0.213 |  | 5.152 |  | 1.441×10^+31^ |  | 1.071 |  |
| group + Gender + Age + ICAR_measure_prop |  | 0.050 |  | 0.169 |  | 3.864 |  | 1.142×10^+31^ |  | 1.496 |  |
| group + Age + ICAR_measure_prop |  | 0.050 |  | 0.138 |  | 3.053 |  | 9.354×10^+30^ |  | 1.085 |  |
| group + Gender + Age + ICAR_measure_prop + group ✻  Gender |  | 0.050 |  | 0.087 |  | 1.807 |  | 5.868×10^+30^ |  | 1.929 |  |
| Gender + Age |  | 0.050 |  | 5.099×10^-13^ |  | 9.689×10^-12^ |  | 3.446×10^+19^ |  | 2.568 |  |
| Age |  | 0.050 |  | 3.470×10^-13^ |  | 6.593×10^-12^ |  | 2.345×10^+19^ |  | 0.001 |  |
| group + Gender + Age |  | 0.050 |  | 2.520×10^-13^ |  | 4.788×10^-12^ |  | 1.703×10^+19^ |  | 3.233 |  |
| group + Gender + ICAR_measure_prop + group ✻  Gender |  | 0.050 |  | 1.285×10^-13^ |  | 2.441×10^-12^ |  | 8.683×10^+18^ |  | 1.384 |  |
| group + Gender + Age + group ✻  Gender |  | 0.050 |  | 1.117×10^-13^ |  | 2.123×10^-12^ |  | 7.549×10^+18^ |  | 5.937 |  |
| group + Age |  | 0.050 |  | 7.052×10^-14^ |  | 1.340×10^-12^ |  | 4.765×10^+18^ |  | 1.046 |  |
| group + Gender + ICAR_measure_prop |  | 0.050 |  | 3.461×10^-15^ |  | 6.576×10^-14^ |  | 2.339×10^+17^ |  | 1.116 |  |
| group + ICAR_measure_prop |  | 0.050 |  | 5.393×10^-16^ |  | 1.025×10^-14^ |  | 3.644×10^+16^ |  | 0.759 |  |
| ICAR_measure_prop |  | 0.050 |  | 8.336×10^-22^ |  | 1.584×10^-20^ |  | 5.633×10^+10^ |  | 9.117×10^-4^ |  |
| Gender + ICAR_measure_prop |  | 0.050 |  | 3.639×10^-22^ |  | 6.914×10^-21^ |  | 2.459×10^+10^ |  | 0.931 |  |
| group + Gender + group ✻  Gender |  | 0.050 |  | 6.146×10^-25^ |  | 1.168×10^-23^ |  | 4.153×10^+7^ |  | 7.188 |  |
| group + Gender |  | 0.050 |  | 2.349×10^-26^ |  | 4.462×10^-25^ |  | 1.587×10^+6^ |  | 1.953 |  |
| group |  | 0.050 |  | 1.070×10^-27^ |  | 2.033×10^-26^ |  | 72293.619 |  | 3.725×10^-7^ |  |
| Gender |  | 0.050 |  | 1.610×10^-32^ |  | 3.059×10^-31^ |  | 1.088 |  | 0.019 |  |
|  | | | | | | | | | | | |

**Experiment 4: Social and Non-social Attentional Orientation (Arrow/Gaze Cueing Task)**

Note: As there were two dependent variables, alpha was set to .025.

*Errors*. There was no main effect of group (*F*(1, 967) = 0.006, *p* = .938, η_p_^2^ = 6.277 x 10^-6^). Age had a negative relationship with error rate (*F*(1, 967) = 49.904, *p* < .001, η_p_^2^ = .049). Cue type had a significant main effect (*F*(1, 967) = 45.941, *p* < .001, η_p_^2^ = .045), such that errors were more common following arrow (2.6%) than gaze (1.3%) trials. Cue validity also had a main effect (*F*(1, 967) = 117.958, *p* < .001, η_p_^2^ = .109), such that errors were more common following invalid (3.1%) than valid (0.9%) cues. Cue type interacted with cue validity (*F*(1, 967) = 74.547, *p* < .001, η_p_^2^ = .072), such that the difference in error rates between valid and invalid arrow cues (4.1%) was greater than the difference between valid and invalid gaze cues (0.3%), *F*(1, 972) = 292.297, *p* < .001, η_p_^2^ = .231. Age interacted with cue type (*F*(1, 967) = 23.710, *p* < .001, η_p_^2^ = .024), validity (*F*(1, 967) = 54.194, *p* < .001, η_p_^2^ = .053), and in a three-way interaction with cue type and cue validity (*F*(1, 967) = 43.110, *p* < .001, η_p_^2^ = .043), such that the magnitude of the disparity between arrow and gaze cueing effects correlated negatively with age (*r* = -0.224, *p* < .001). All other *F* ≤ 4.086; all other *p* ≥ .044.

| Supplementary Table 5: Descriptive statistics demonstrating numerically larger cueing effects for both cue types in the arrow/gaze cueing task.  **Descriptives** | | | | | | | | | | | | | | | |
| --- | --- | --- | --- | --- | --- | --- | --- | --- | --- | --- | --- | --- | --- | --- | --- |
| **Type** | | **Validity** | | **Group** | | **N** | | **Mean** | | **SD** | | **SE** | | **Coefficient of variation** | |
| Arrow |  | Valid |  | autism |  | 557 |  | 377.319 |  | 64.952 |  | 2.752 |  | 0.172 |  |
|  |  |  |  | no autism |  | 416 |  | 335.364 |  | 53.752 |  | 2.635 |  | 0.160 |  |
|  |  | Invalid |  | autism |  | 557 |  | 413.477 |  | 65.919 |  | 2.793 |  | 0.159 |  |
|  |  |  |  | no autism |  | 416 |  | 368.492 |  | 51.685 |  | 2.534 |  | 0.140 |  |
| Gaze |  | Valid |  | autism |  | 557 |  | 384.722 |  | 64.330 |  | 2.726 |  | 0.167 |  |
|  |  |  |  | no autism |  | 416 |  | 344.277 |  | 53.112 |  | 2.604 |  | 0.154 |  |
|  |  | Invalid |  | autism |  | 557 |  | 388.474 |  | 64.638 |  | 2.739 |  | 0.166 |  |
|  |  |  |  | no autism |  | 416 |  | 345.907 |  | 50.846 |  | 2.493 |  | 0.147 |  |
|  | | | | | | | | | | | | | | | |

Reference:

Agelink van Rentergem, J. A., I. E. Vermeulen, P. R. Lee Meeuw Kjoe, and S. B. Schagen. 2020. “Computational Modeling of Neuropsychological Test Performance to Disentangle Impaired Cognitive Processes in Cancer Patients.” JNCI Journal of the National Cancer Institute 113, no. 1: 99–102.

1. We also conducted this analysis with the number of errors divided by the number of nodes on each respective part (as per our pre-registration), and the results did not change. We report the results of analyzing the total number of errors per part here instead for the sake of more easily interpretable means and figures. [↑](#footnote-ref-1)
